# Weekend Light Shifts Evoke Persistent *Drosophila* Circadian Neural Network Desynchrony

**DOI:** 10.1523/JNEUROSCI.3074-19.2021

**Published:** 2021-06-16

**Authors:** Ceazar Nave, Logan Roberts, Patrick Hwu, Jerson D. Estrella, Thanh C. Vo, Thanh H. Nguyen, Tony Thai Bui, Daniel J. Rindner, Nicholas Pervolarakis, Paul J. Shaw, Tanya L. Leise, Todd C. Holmes

**Affiliations:** ^1^Department of Physiology and Biophysics, University of California, Irvine, Irvine, California 92697; ^2^Center for Complex Biological Systems, University of California, Irvine, Irvine, California 92697; ^3^Department of Anatomy and Neurobiology, Washington University in St. Louis, St. Louis, Missouri 63110; ^4^Department of Mathematics and Statistics, Amherst College, Amherst, Massachusetts 01002

**Keywords:** circadian rhythm, *Drosophila*, learning, memory, neural circuits, sleep

## Abstract

We developed a method for single-cell resolution longitudinal bioluminescence imaging of PERIOD (PER) protein and TIMELESS (TIM) oscillations in cultured male adult *Drosophila* brains that captures circadian circuit-wide cycling under simulated day/night cycles. Light input analysis confirms that CRYPTOCHROME (CRY) is the primary circadian photoreceptor and mediates clock disruption by constant light (LL), and that eye light input is redundant to CRY; 3-h light phase delays (Friday) followed by 3-h light phase advances (Monday morning) simulate the common practice of staying up later at night on weekends, sleeping in later on weekend days then returning to standard schedule Monday morning [weekend light shift (WLS)]. PER and TIM oscillations are highly synchronous across all major circadian neuronal subgroups in unshifted light schedules for 11 d. In contrast, WLS significantly dampens PER oscillator synchrony and rhythmicity in most circadian neurons during and after exposure. Lateral ventral neuron (LNv) oscillations are the first to desynchronize in WLS and the last to resynchronize in WLS. Surprisingly, the dorsal neuron group-3 (DN3s) increase their within-group synchrony in response to WLS. *In vivo*, WLS induces transient defects in sleep stability, learning, and memory that temporally coincide with circuit desynchrony. Our findings suggest that WLS schedules disrupt circuit-wide circadian neuronal oscillator synchrony for much of the week, thus leading to observed behavioral defects in sleep, learning, and memory.

## Significance Statement

The circadian clock controls numerous aspects of daily animal physiology, metabolism and behavior. Much of our understanding of circadian circuit-level oscillations stem from *ex vivo* imaging of mammalian suprachiasmatic nucleus (SCN) brain slices. Humans regularly subject themselves to weekday/weekend light shifts (WLSs) but the effects of phase-shifting light signals cannot be measured in SCN. We measured circuit-level circadian responses to a weekday/WLS protocol in light-sensitive *ex vivo Drosophila* whole-brain preparation that shows temporal coincidence to circadian behavioral events. Robust subcircuit-specific oscillator desynchrony/resynchrony responses to light coincide with functional defects in learning and memory, and sleep pattern disruption *in vivo*. Our results reflect that WLS causes circadian-circuit desynchronization and correlates with disrupted cognitive and sleep performance.

## Introduction

The modern workplace results in individuals across the world subjecting themselves to phase-advancing light shifts on Monday morning after staying up later during weekends starting on Friday, with phase-delaying light signals persisting throughout the weekend. Disruptions to circadian rhythmicity is linked to serious pathophysiological illnesses such as heart disease, diabetes, and cancer (Moore-Ede et al., 1983; Hastings et al., 2003; Scheer et al., 2009; Papagiannakopoulos et al., 2016). The effects of acute light shifts, like WLS, are not known for any circadian neural circuit.

PERIOD (PER) protein cycling imaging in suprachiasmatic nucleus (SCN) provides detailed functional data on the central neural circuits that govern circadian rhythms and forms the basis for interpreting the linkage between the timing of clock cycling and circadian physiological outputs in mammals (Welsh et al., 1995; Hamada et al., 2001; Yamaguchi et al., 2003; Evans et al., 2013; Azzi et al., 2017). Longitudinal optical or electrical recording of large numbers of neurons in SCN slices no longer driven by light cues in *ex vivo* conditions shows that free-running between-oscillator phases are complex and relatively desynchronized (Quintero et al., 2003; Schaap et al., 2003; Yamaguchi et al., 2003). Clock cycling in mammalian SCN slices can be longitudinally imaged, but their ongoing direct responses to environmental light signals cannot be studied this way because of the absence of physiological light input into the SCN in *ex vivo* preparations (Welsh et al., 1995; Moga and Moore, 1997; Hamada et al., 2001; Evans et al., 2013). Acute physiological responses can be measured in SCN *ex vivo* slices after stimulating the retinohypothalamic tract with a stimulating electrode (Irwin and Allen, 2007).

The primary light input mechanism for the fly circadian neural circuit is via the blue-light sensitive photoreceptor CRYPTOCHROME (CRY) expressed in roughly half of the fly circadian neurons (Stanewsky et al., 1998; Benito et al., 2008; Yoshii et al., 2008; Fogle et al., 2011), including all small-lateral ventral neurons (s-LNvs), all large-LNvs (l-LNvs), 50% of lateral-dorsal neurons (LNds), and dorsal neurons-1 (DN1). A secondary photoreceptor responsive to broad light spectra, Rhodopsin-7 (Rh7), is expressed in *Drosophila* circadian neurons, in photoreceptors (Kistenpfennig et al., 2017; Ni et al., 2017; Sakai et al., 2017), and external, opsin-based photoreceptors provide redundant photic input (Helfrich-Förster et al., 2001; Li et al., 2018). We took advantage of cell autonomous photoreceptors in many central brain circadian neurons to develop an imaging system that measures bioluminescence of PER oscillation at single-cell resolution for up to 11 d in a whole-brain culture system (Roberts et al., 2015, 2016). Bioluminescence imaging of highly light-sensitive circadian neurons avoids all possibility of light contamination caused by fluorescence excitation and single cell resolution multiday imaging verifies circadian rhythmic activity (Hege et al., 1997; Roberts et al., 2015, 2016).

Our earlier work reveals circadian-circuit-wide response of PER cycling in constant darkness (DD) to a phase-advancing light pulse at the single-cell-resolution. The response consists of systematic desynchrony followed by resynchronization of PER cycling. This unique behavior varies between the different neuronal subclasses of the circadian circuit observed under DD conditions immediately after of the light pulse (Roberts et al., 2015, 2016). Here, we use *Drosophila* brains maintained under simulated 24-h daytime and nighttime cycles to study neural circuit response to WLS. The timing of the WLS schedule resembles the weekend/weekday light shifts experienced by many humans. Furthermore, we verify circuit imaging with *in vivo* behavioral analysis by testing *cry^b^* mutants and environmental disruption of the circadian clock by constant light (LL).

Both mammalian and fly rhythms rely on circadian pacemaker circuit networks coupled by peptide and small-molecule neurotransmitters. In *Drosophila*, this includes the neuropeptide, pigment-dispersing factor (PDF), which coordinates circadian phase (Renn et al., 1999; Maywood et al., 2006; Wu et al., 2008; Johard et al., 2009; Shafer and Yao, 2014; Liang et al., 2016*;* Jones et al., 2018). Motivated by the many functional similarities between mammalian and fly circadian circuitry, we investigated the effects of light shifts on circadian rhythmicity using *Drosophila*.

## Materials and Methods

### Experimental design

#### Behavioral analysis of day-night entrainment

TriKinetics *Drosophila* activity monitor (DAM) system was employed to record the locomotor activity of adult wild-type (W^1118^[5905]), transgenic XLG-PER-Luc, and *ptim*-TIM-Luc flies (Roberts et al., 2015, 2016; Lamba et al., 2018). Individual flies were placed in 5 mm Pyrex glass tubes with fly food on one end, and a cotton plug on the other. Each experiment was run with either 32 or 64 adult male flies. The fly-containing tubes are mounted in a DAM5 DAM (TriKinetics) which records the number of infrared beam crossings over time, as a measure of activity. Flies are first entrained under standard 12/12 h light/dark (12/12LD) conditions for ≥3 d. Following entrainment, flies are then exposed to either the LD strobe protocol that for each hour of “light” 15minLight:45minDark is repeated ever hour for 12 h superimposed over a 12/12LD; skeleton photoperiod (SPP); or standard 12/12LD light protocols with consistent phases and a consistent (white light intensity: of 1.1 mW/cm^2^) for 8 d. The LD strobe protocol is performed by dividing the 12 h of daytime entrainment into 12 1-h cycles of short, intermittent light-dark exposures followed by 12 h of nighttime darkness. The 15-min SPP protocol is performed by applying a short light pulse at the transition times of expected lights on (simulated dawn) and lights off (dusk) based on the previous standard entrainment. Initial optimization tests for LD strobe and SPP protocols were determined with light pulse durations of either 5, 15, or 30 min using highest behavioral circadian rhythmicity under subsequent DD conditions as comparison criteria (Table 1). Following the 8 d of entrainment by either 12/12LD, LD strobe, or SPP, we examined the free-running circadian activity of the flies for ≥3 d under DD.

**Table 1.** Quantification of behavioral entrainment by LD strobe and SPP

		W1118 [5905]			XLG-PER-Luc			TIM-Luc		
	*n*	% Rhythmic	Period	*n*	% Rhythmic	Period	*n*	% Rhythmic	Period
Std. entrainment									
LD	60	96.7	23.9	57	100	23.9	61	98.4	24
DD	57	100	23.5	54	90.7	23.3	56	96.4	23.8
30L30D LD strobe									
LD	54	100	23.8	46	95.7	23.8	49	98	23.8
DD	53	100	23.5	45	64.4	23	47	89.4	23.8
15L45D LD strobe									
LD	61	100	23.7	41	97.6	23.9	37	94.6	23.9
DD	60	100	23.5	36	75	23.2	28	89.3	23.7
5L55D LD strobe									
LD	56	98.2	23.6	58	98.3	23.8	56	96.4	23.7
DD	51	94.1	23.4	54	74.1	23.3	52	92.3	23.5
30-min SPP									
LD	61	98.4	23.9	56	94.6	23.9	58	79.3	23.8
DD	59	98.3	23.6	51	82.4	23.3	55	90.9	23.3
15-min SPP									
LD	62	100	23.6	55	74.5	23.8	60	88.3	23.9
DD	61	98.4	23.4	50	54	23.7	59	76.3	23.7
5-min SPP									
LD	63	100	23.6	62	95.2	23.7	57	94.7	23.7
DD	63	98.4	23.3	62	87.1	23.1	56	94.6	23.5

FaasX was used for analysis of behavioral experiments. Wild-type (W1118[5905]) and XLG-luc flies were exposed to 5 d of entrainment (LD) by either LD strobe or SPP protocols with 30-, 15-, or 5-min pulses. Following entrainment, flies were maintained in DD for 5 d. Cycle-P was used to quantify measures of period length and the percentage of rhythmic flies using 15-min bins of individual fly locomotor activity. Flies were scored as rhythmic by χ^2^ periodogram analysis if they met the following criteria: power ≥ 40, width ≥ 4 h, and period length of 24 ± 8 h.

#### Bioluminescence imaging

Custom bioluminescence set up is designed and built by Logan Roberts with David Callard, and Jeff Stepkowski (Stanford Photonics) and Todd Holmes. Bioluminescence set up includes custom light filters, LED light set up by Prizmatix, a retooled and light-tight black box, and custom temperature control maze. Bioluminescence imaging is performed using adult, male XLG-PER-Luc transgenic fly brains (line provided by Ralf Stanewsky, University of Münster, Germany; as descibed in Veleri et al., 2003). *cry^b^*-XLG-PER-Luc (line provided by Ralf Stanewsky, University of Münster, Germany; Harper et al., 2017) and *ptim*-TIM-Luc (provided by Patrick Emery, University of Massachusetts Medical School; Lamba et al., 2018). Transgenic flies (XLG-PER-Luc, *ptim*-TIM-Luc, *cry^b^*-XLG-PER-Luc) are first entrained to ≥3 d of 12/12LD entrainment before dissection. Six whole fly brain explants are dissected and cultured on a single insert per experiment with or without eye photoreceptors attached to the explant (no cuticle tissues included) using a modified version of a previously described protocol (Roberts et al., 2015). The cultured brains are mounted on a stage (Applied Scientific Instrumentation) with automated XYZ movement controlled by the software Piper. The stage is connected to an upright Axio Observer.Z1 Microscope (Zeiss) set in a custom light-tight incubator (designed by Alec Davidson, Morehouse School of Medicine, GA) with temperature maintained at 25 ± 0.5°C. Bioluminescence from the cultured whole brains is collected by a Zeiss 5× (NA = 0.25) objective and transmitted directly to a MEGA-10Z cooled intensified CCD camera (Stanford Photonics) mounted on the bottom port of the microscope. The XY position of the samples is manually set using bright-field illumination. The optimal z-plane of focus for bioluminescence imaging is obtained by performing 10 Z-steps at 40- to 50-µm intervals with 5- to 10-min exposures. Experimental bioluminescence imaging of the samples is obtained with 15-min exposures at 30 fps for ≥11 d of recording at single-cell resolution during the hourly dark phase of the LD strobe protocol. Light exposure and entrainment are performed using an LD strobe protocol with the 12 h of daytime entrainment divided into 12 consecutive cycles of a 15-min light pulse and 45 min of darkness, followed by 12 h of DD (hereby referred to as 15L45D/LD strobe). Bioluminescence imaging under LL entrainment uses the same parameters of LD strobe but differs in that there is no 12 h of DD. For the entirety of the LL period, brains are exposed to 8 d of consecutive cycles of 15-min light pulse, followed by 45 min of darkness, until the onset of DD. Images are collected by Piper (Stanford Photonics) and averaged into 45-min bins by ImageJ before using MetaMorph (Molecular Devices), Microsoft Excel and custom MATLAB scripts to measure circadian parameters of bioluminescence cycling with single cell resolution. Only experiments with all six brain explants still healthy, contamination-free, adhering to the insert substrate, and exhibiting bioluminescence for ≥11 d are used for analysis. For counting signals from regions of interest (ROIs), the script factors in the background ROI for that one specific frame. Because the values of the background vary per frame, the calculated photons can range between positive and negative values.

#### Simulating day-night entrainment and WLSs *ex vivo*

To establish baseline measurements of day-night entrainment, one group (referred to as the control group) consists of whole-brain explants exposed to the 15L45D LD strobe schedule that simulates 12/12LD entrainment for 8 d with no phase shifts followed by ±3 d of DD. Stable white light exposure [30 µW/cm^2^, as performed in our previous published work (Roberts et al., 2015, 2016) using a mic-LED (Prizmatix)] is set to provide a stable light intensity with automated timing set via TTL input from Piper (Stanford Photonics). During intervals of light exposures, the CCD camera is protected by a mechanical shutter controlled via TTL input from Piper to allow for semi-continuous imaging. For samples exposed to a WLS protocol (referred to as WLS), the first three recorded “weekdays” (all with the same phase for a simulated “Wednesday” to “Friday”) have parallel phases with the control group. This is followed by a 3-h phase delay on the evening of the third recorded day (simulated “Friday night”) followed by two “weekend” days but with no phase shift (simulated “Saturday” through “Sunday”). Whole-brain explants are exposed to a phase advance of 3 h on the morning of the sixth day of recording (simulated “Monday” morning) with no phase shifts for the following simulated weekdays (Monday through Wednesday). Finally, explants are placed in DD for ≥3 d. Three-hour phase shifts were used because they correspond with social behaviors commonly observed in the general populace regarding WLSs and have been linked to negative health effects. LED light exposure and brain imaging are automated via TTL input through the Piper software provided by Stanford Photonics (control group = 208 players, WLS group = 214 players).

#### Processing of bioluminescence images

Cosmic rays are removed in real-time using the Piper cosmic ray filter set to discriminate the sum of all pixel values above 800 and reject frames that are >3 SDs over the running average (run over 30 frames). ImageJ is used to generate images with bioluminescence images averaged over 45-min intervals. These images were then further processed using MetaMorph as described in previously work (Roberts et al., 2015, 2016). Briefly, noise from dark current and cosmic rays were removed by using a running minimum algorithm to generate new images constructed from pairs of sequential images using the minimum values of each pixel from the two images. MetaMorph was used to generate a stack of images for each experiment with average luminescence intensity over time measured for ROIs that were manually defined based on a previous protocol (Roberts et al., 2015). ROIs were classified into circadian neuron groups (color-coded: red = s-LNv, yellow = l-LNv, orange = LNd, blue = DN1, green = DN3) based on consistent and classically recognized anatomic locations. Raw bioluminescence data were then processed by Microsoft Excel and was adjusted for background noise and convert raw luminescence over time to photons-per-minute as previously described (Roberts et al., 2015, 2016). Circadian parameters were analyzed for 11-d recordings using modified versions of previously described MATLAB scripts with the first 12 h excluded because of initially high amplitude and highly variable bioluminescence following dissection and addition of luciferin (Roberts et al., 2015). Between circadian neuronal cell groups, variable bioluminescence persists for several days after dissection. These records are retained to show re-emergence of highly synchronized between circadian cell group rhythms after several days in culture. This is in strong agreement with anti-PER immunocytochemical (ICC) “snapshots” of highly synchronous *in vivo* fly brain PER cycling measured in flies maintained in LD over 24 h (Zerr et al., 1990).

#### Learning and memory assay

Flies were evaluated for the effects of the WLS on both sleep and short-term memory (STM; Seugnet et al., 2009). Approximately 6-d-old male Canton-S (Cs) flies were used to assess STM using the aversive phototaxic suppression (APS) assay. Before being tested for STM, flies are examined to determine whether they exhibit normal photosensitivity and quinine photosensitivity. This step is used to ensure that the changes to sensory thresholds are true changes in associative learning. Photosensitivity is evaluated using a T-maze with one lightened and darkened chambers that appear equal on either side. Flies must make photopositive choices to be considered for post-WLS evaluation of STM. Quinine sensitivity index (QSI) is achieved by determining the duration a fly stay on a side of a T-maze without quinine, as opposed to one side with the aversive stimuli, during a 5-min period (Seugnet et al., 2009). The learning test to evaluate STM used the APS assay. Flies were subjected to WLS schedule used for bioluminescence and behavior experiments. Flies were tested on the subjective Tuesday of the WLS schedule, 2 d after 3-h phase shifts experienced during the weekend. Flies are individually placed in a T-maze and allowed to choose between a lighted or darkened chamber for over 16 trials. Flies that do not display phototaxis during the first block of four trials are excluded from further trials. During the 16 trials, flies learn to avoid the lighted chamber paired with aversive stimulus (Seugnet et al., 2009). The performance index is calculated as the percentage of the times the fly choses the dark vial during the last four trials of the 16-trial test. STM is defined as selecting the dark vial on two or more occasions during the final four tests.

### Statistical analysis

#### Quantification of locomotor activity

We used the FaasX (M. Boudinot and F. Rouyer, Center National de la Recherche Scientifique) program for analysis of locomotor activity recorded by the automated TriKinetics DAM system. Cycle-P was used to quantify period length, amplitude, and rhythmicity using 15-min bins of individual fly locomotor activity. Individual fly rhythmicity is defined rhythmic based on χ^2^ periodogram analysis with the following criteria (high frequency filter on): power ≥40, width ≥4 h and period length of 24 ± 8 h. Double-plotted actogram graphs were generated by the software ClockLab (Actimetrics) showing normalized activity over 1-min intervals.

#### Quantification of circadian oscillator dynamics

Custom MATLAB scripts (version 8.2) were employed to analyze real-time bioluminescence recordings for quantification of order parameter, goodness-of-sine-fit, amplitude, period and phase. The order parameter “R” was used to quantify the synchrony of phase, period and waveform for each circadian neuron subgroup and for “all cells” (combined set across all subgroups), as defined in Roberts et al. (2015). This definition uses the entire detrended time series, rather than phase estimates, applied to 24-h-long segments bounded by time of lights on under the control LD cycle. Statistical significance of R values was determined by applying a randomization procedure calculating the R value for randomly selected subsets of cells from the combined control and WLS conditions, using the number of cells in the smallest cluster. Each randomization procedure was repeated 5000 times to provide 95% and 99% confidence intervals under the null hypothesis of no difference in R (R_WLS_ – R_CTRL_) between cells exposed to WLS and cells in control conditions with no phase shifts. For R_WLS_ – R_CTRL_ statistical comparisons, the darker shaded band indicates 95% confidence level for significance, lighter shaded band indicates 99% confidence level for significance; within the shaded bands indicates no significant differences while exiting the shaded bands indicate significant differences. The discrete wavelet transform (DWT) was used in combination with sine-fit estimates of 2-d sliding windows to provide circadian measures of rhythmicity, period, amplitude, and phase. Oscillators were deemed “reliably rhythmic” if their period was 24 ± 8 h, amplitude rose above a baseline noise value (SD of the DWT component associated with periods shorter than 4 h), and their goodness-of-sine-fit measure was ≥0.82 as described previously (Roberts et al., 2015). Circular phase plots were generated using the midpoint of 2-d sliding windows with an inner circle showing the α = 0.05 threshold for the resultant vector for the Rayleigh test. Circular plots were standardized with ZT0 set equal to the overall mean phase of all cells in the control condition on day 3 when entrainment is most stable. The absence of an oscillator’s plots at certain time points indicates that the oscillator’s rhythmicity did not meet the criteria for reliably rhythmic cells and was thus too dampened to reliably measure. Note that no cells were discarded when calculating R values; the reliably rhythmic criteria were only applied when reporting phase values.

#### Non-linear embedded phase estimates used to generate phase ensemble animations and validate sine-fit estimates

A time delay embedding protocol was used to confirm that circadian parameter s was reliably measured using sine-fits of wavelet detrended time series. Phase estimates were determined by the polar angle of time series that were embedded in a higher dimension via a 6-h lag resulting in oscillations circling the origin. Phase plots generated using this nonlinear embedded phase analysis confirmed the same patterns of oscillator dynamics observed in plots generated using sine-fit calculations. Non-linear embedded phase estimates were also used to generate phase ensemble animations as previously described (Roberts et al., 2016). Dynamic changes in phase and amplitude were displayed at three levels of resolution: whole circadian neuron network (Movie 4), individual neuronal subgroups (Movie 5), and single neuron oscillators (Movie 6). In Movies 4, 5, the polar angle of each disk represents the relative phase shift in control and WLS conditions. The size and proximal distance of the disks from the center of the circle represents amplitude (i.e., damping is indicated by a shrinking disk drifting toward the center). In Movie 6, the polar angle of each disk indicates the phases of individual neuron oscillators while amplitude is again reflected by the size of the disks and proximal distance from the center.

#### Fly sleep quantification

Fly activity data were binned into 60-min time sections. Following binning, activity data were translated to run length encoding and any run of zero activity for five or greater minutes was scored as sleep. Each fly’s total amount of sleep per bin was totaled, and the resultant matrix contained the total amount of sleep by fly per 60-min increments for the length of the experiment. Flies that died during the course of the experiment registered very long strings of zero activity and were manually removed to prevent over counting sleep amounts. Custom MATLAB scripts were used to create sleep heat maps. Because of sleep distributions that are non-normal (floor and ceiling effects), a Wilcoxon Rank Sum test was used for the difference in median with α = 0.01.

## Results

### Development of LD strobe to simulate day-night entrainment

**Figure 1.**
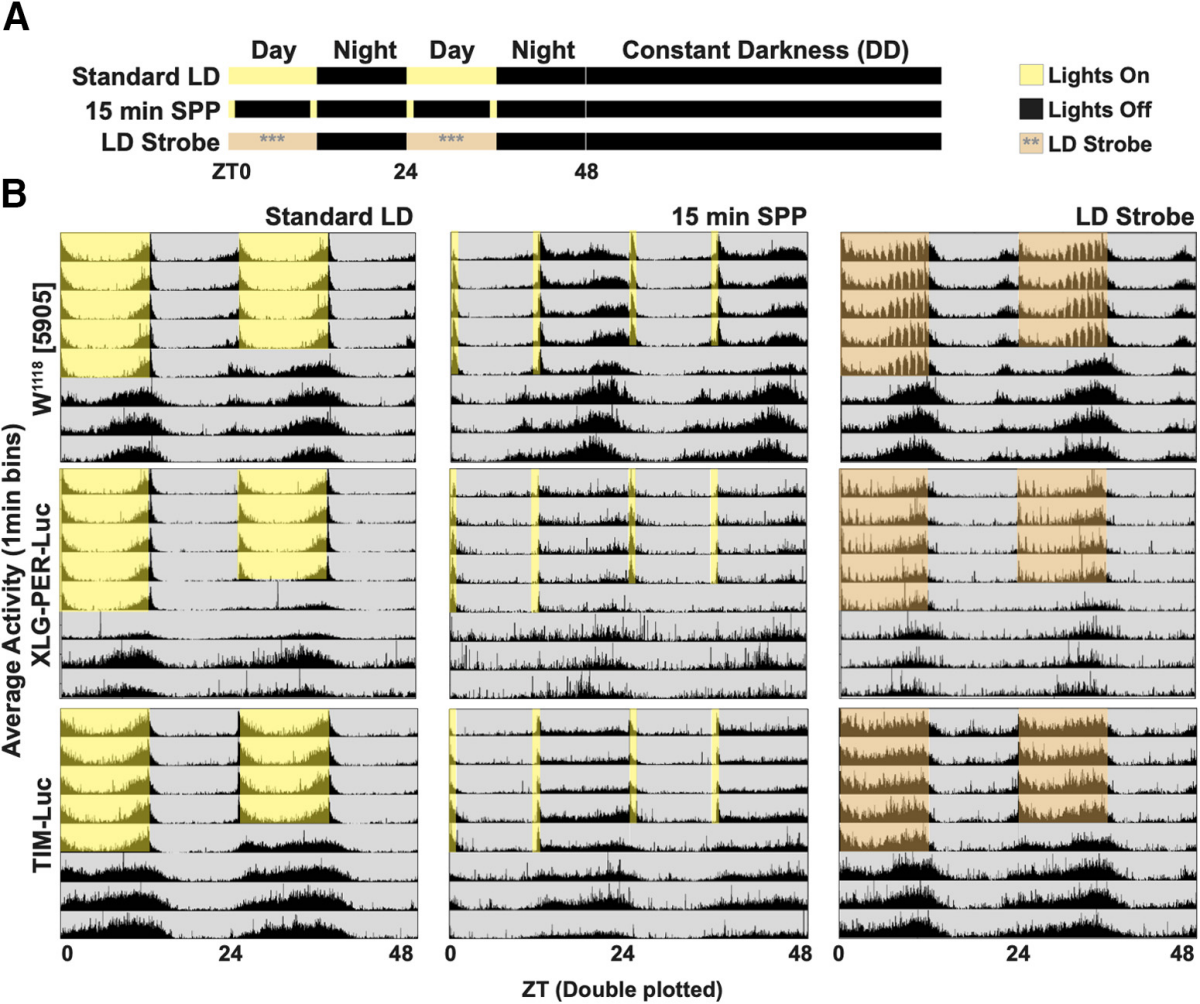
Day-night entrainment of locomotor activity by LD strobe and SPP. Averaged double-plotted locomotor activities of adult *Drosophila* for 5 d of entrainment by various light protocols followed by 3 d of DD. ***A***, All flies are entrained to ≥3 d of 12 h:12 h LD before exposure to control standard LD (***A***, first row), 15 min SPP (***A***, middle row; yellow bars indicate 15 min of light exposure at caps of each 12-h day), or LD strobe (***A***, lower row; orange shade indicates 15 min of light followed by 45 min of dark for every hour of the 12-h day). Yellow or orange shade indicates windows of light exposure, black indicates lights off. ***B***, Behavior actograms for three fly genotypes (W1118[5905], upper row; XLG-PER-Luc, middle row, and *ptim*-TIM-Luc, lower row) in three different entrainment schedules (standard LD, left column; 15 min SPP, middle column; LD strobe, right column). All entrainment schedules involve 5 d of respective light/dark regimes (yellow or orange shade/gray shade), followed by 3+ d of DD (gray shade). See Table 1 for number of flies used.

Light is the primary environmental cue for circadian entrainment (Hall, 2005). *Drosophila* brains are directly light sensitive because of the expression of cell-autonomous, short-wavelength, light-sensitive photoreceptors. *Drosophila* process external environmental light signals via CRY and Rh7, along with redundant photic input from the eyes and other opsin-based external photoreceptors. The direct sensitivity of the fly brain to light input enables the measurement of physiological photic entrainment using real-time bioluminescence recordings of entire cultured brains (Roberts et al., 2015, 2016). In addition to long light exposure, circadian cycles can be entrained using short pulses of light, referred to as SPP (Pittendrigh and Minis, 1964; Pittendrigh and Daan, 1976). In SPP, light pulses flank the beginning and end of the simulated daytime, which is then followed by long periods of DD that simulate nighttime. This suggests that obtaining bioluminescence images during simulated daytime is feasible. However, compared with the robust circadian behaviors under standard 12/12 h light/dark (LD) entrainment (Fig. 1*A*, upper row, *B*, first column), circadian locomotor behaviors in DD following SPP entrainment are relatively weak (Fig. 1*A*, middle row, *B*, middle column) and the two conditions differ across genotypes (Fig. 1*B*, W^1118^[5905] = top row, XLG-PER-Luc = middle row, and *ptim*-TIM-Luc = lower row). As circadian behavior in DD reflects the activity of the free-running clock, we established the criteria that DD behavior, following light schedules used for imaging conditions, must meet statistical criteria for rhythmicity by χ^2^ analysis (see Materials and Methods for details). While multiple light schedules met this statistical criterion, we reasoned that, as light is normally present throughout the day, to simulate day light conditions, we would employ a novel entrainment protocol we refer to as LD strobe for imaging XLG-PER-Luc and *ptim-*TIM-Luc *Drosophila* luciferase lines (Fig. 1*A*, right column; also see Materials and Methods). LD strobe consists of 15-min periods of light followed by 45 min bouts of darkness each hour during the 12-h “day,” then 12 h of darkness during the 12-h “night.” We find that DD behavior following LD strobe is indistinguishable from DD following standard LD (Fig. 1*B*, left vs right column). Thus, LD strobe effectively simulates daytime during 12 h of alternating periods of light and dark for control and -Luc genotypes. The intermittent darkness during the simulated daytime provides the opportunity to capture circadian circuit bioluminescence while simulating a 24-h day.

### Synchronized TIMELESS (TIM)-luciferase rhythmic cycles occur in all major circadian cell groups under LD strobe simulated day/night

**Figure 2.**
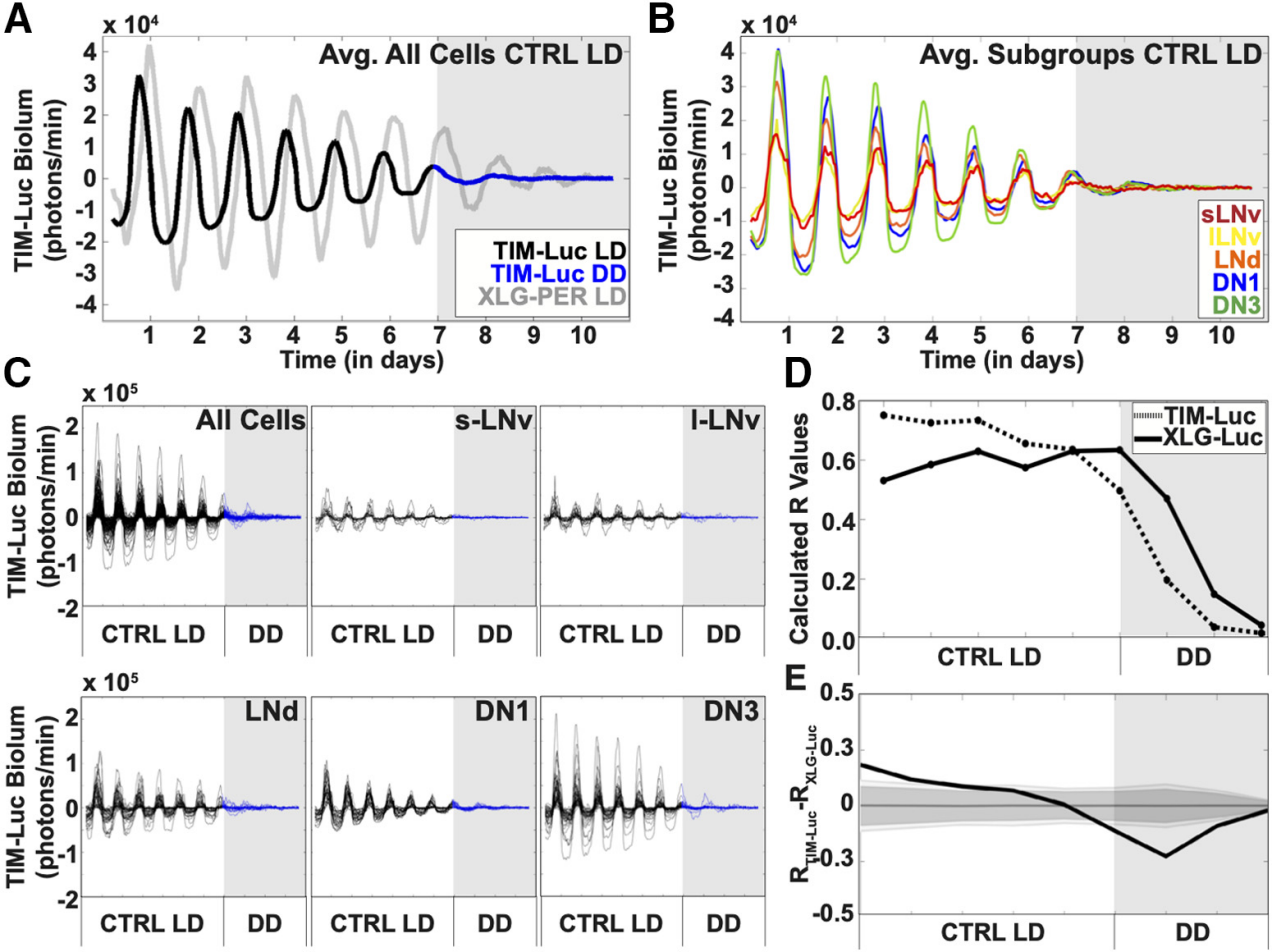
Bioluminescence recording of TIM oscillations in *Drosophila* circadian neurons. Bioluminescence recordings of TIM-Luc in cultured adult *Drosophila* brains (*n* = 6 brains, 159 cells) under control LD (black trace) followed by DD (blue trace, gray shade). XLG-PER-Luc bioluminescence (gray trace) is overlaid for comparison of oscillations between the two genotypes. ***A***, Averaged bioluminescence traces of all TIM-expressing circadian neurons under 7 d of control LD conditions (black trace) followed by DD (blue trace, gray shade). XLG-PER-Luc expression is overlaid for comparison of the two clock proteins (s-LNv = 18 cells, l-LNv = 19 cells, LNd = 18 cells, DN1 = 27 cells, DN3 = 18 cells). ***B***, Averaged bioluminescence traces of TIM expression in circadian neuron subgroups under control LD conditions. Each neuron subgroup is labeled as follows: s-LNv (red), l-LNv (yellow), LNd (orange), DN1 (blue), and DN3 (green) proteins (s-LNv = 15 cells, l-LNv = 24 cells, LNd = 43 cells, DN1 = 35 cells, DN3 = 42 cells). ***C***, TIM bioluminescence traces of individual neurons for all cells (top left panel), s-LNv (top middle panel), l-LNv (top right panel), LNd (lower left panel), DN1 (bottom middle panel), and DN3 (bottom right panel) under control LD conditions. Control LD entrainment involves 7 d of control LD followed by DD (gray shade). ***D***, Calculated synchronization index/order parameter, R values for TIM oscillations (dotted trace) under control LD conditions. XLG-PER-Luc (solid trace), under control conditions is overlaid for comparison. ***E***, Statistical comparisons of overall synchrony between TIM and PER under control LD conditions followed by DD. Difference in order parameter, R, between oscillations of TIM and PER were calculated using a randomization analysis (black trace). Dark gray and light gray zones indicate 95% and 99% confidence bands, respectively, under the null hypothesis that there is no difference in order parameter, R, between the oscillations of TIM and PER under control LD conditions; values that fall outside the dark band are statistically significant.

We compared oscillations of *period* and *timeless* promoter-driven luciferase signals from the transgenic lines: XLG-PER-Luc (Veleri et al., 2003; Roberts et al., 2015) and *ptim*-TIM-Luc (Lamba et al., 2018). We imaged whole brains under the LD strobe light schedule to capture bioluminescence under a simulated daytime/nighttime. The averaged bioluminescence signals from all clock neurons of XLG-PER-Luc and *ptim*-TIM-Luc are highly rhythmic and synchronous under simulated day/night (Fig. 2*A*) but exhibit notable differences. We found that non-clock neuronal background bioluminescence of *ptim*-TIM-Luc (Movie 1) is remarkably lower than that for XLG-PER-Luc (Movie 2) allowing for more efficient single-cell tracking on cultured brains. Comparison of bioluminescence traces show that the phase of *ptim*-TIM-Luc is advanced a few hours (∼3 h) and shows lower amplitudes relative to XLG-PER-Luc (Fig. 2*A*). The waveform of *ptim*-TIM-Luc is asymmetric with a broader and shallow trough compared with the peak, while XLG-PER-Luc exhibits symmetric peaks and troughs for its waveform (Fig. 2*A*). The broader and shallow trough compared with the peak asymmetric in ptim-TIM-Luc measurements may be because of signal below the threshold of detection at the trough, or photic TIM degradation, or other unknown factors. The waveform characteristics seen in the averaged clock neuron bioluminescence signals of *ptim*-TIM-Luc are seen also in the averaged bioluminescence signals of the individual subgroups: the s-LNvs, l-LNvs, the LNds, and the DN1 and DN3 subgroups (Fig. 2*B*,*C*). High levels of synchrony of phase, period, and waveform between individual neurons among subgroups are observed and are shown by individual superimposed records (Fig. 2*C*) and quantified by order parameter R, which factors phase, period and coherence to measure levels of synchrony (Fig. 2*D*). Statistical comparison of order parameter R between XLG-PER-Luc and *ptim*-TIM-Luc exhibit few significant differences during simulated day/night; however, synchrony and waveform amplitude drop quickly for *ptim*-TIM-Luc in DD conditions (Fig. 2*E*). The asymmetric waveform seen for *ptim*-TIM-Luc may reflect biological signal or more likely, because of overall lower signal amplitude relative to XLG-PER-Luc, we may not be able to detect the lowest *ptim*-TIM-Luc signals. Because we cannot yet distinguish between these possibilities, we focused the remainder of our analysis using XLG-PER-Luc whole-brain imaging.

Movie 1. Raw time-lapse bioluminescence recordings of adult TIM-Luc *Drosophila* whole-brain explants under CTRL LD entrainment. Six whole-brain culture explants of male adult TIM-Luc fly brains under control LD entrainment schedule (7 d of control LD strobe, no shifts, followed by 3 d of DD). See Materials and Methods for details on obtaining bioluminescence image data for analysis. 10.1523/JNEUROSCI.3074-19.2021.video.1

Movie 2. Raw time-lapse bioluminescence recordings of adult XLG-PER-Luc *Drosophila* whole-brain explants cultured for 11 d. Left, Six whole-brain culture explants maintained in control conditions (LD strobe with no phase shift) for 9 d followed by 2 d of DD. Right, Six whole-brain culture explants exposed to WLSs for 9 d followed by transfer to DD for the final 2 d. See Materials and Methods for more details. 10.1523/JNEUROSCI.3074-19.2021.video.2

### Per cycling in the circadian circuit under simulated LD and in response to light shifts does not differ with the presence or absence of eyes

**Figure 3.**
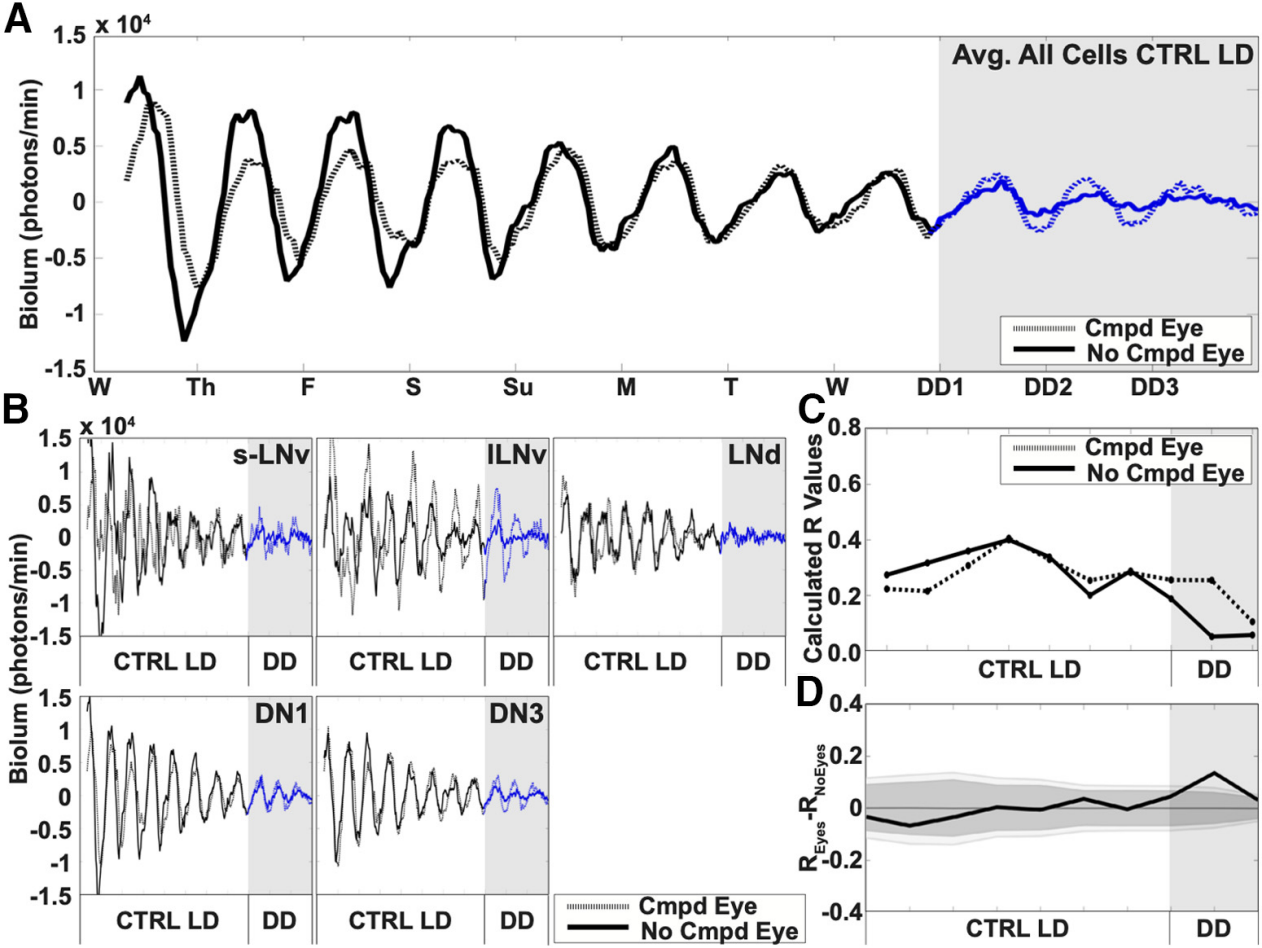
Circadian neuron subgroups are light entrained with either the presence or absence of the compound eyes. Averaged 11-d bioluminescence recordings of cultured *Drosophila* brains with compound eyes attached (*n* = 3 brains, 70 cells, dotted trace) or completely removed (*n* = 3 brains, 63 cells, solid trace) reported by PERIOD from XLG-PER-Luc flies. ***A***, Control LD conditions simulate standard 12/12LD entrainment spanning one week without phase shifts for cultured brains with compound eyes (sLNv *n* = 6, lLNv *n* = 5, LNd *n* = 7, DN1 *n* = 30, DN3 *n* = 22) and brains with compound eyes removed (sLNv *n* = 7, lLNv *n* = 7, LNd *n* = 7, DN1 *n* = 29, DN3 *n* = 13) followed by DD (gray shade). ***B***, Averaged bioluminescence traces for individual neuronal subgroups comparing oscillators in brains with or without attached compound eyes. ***C***, Calculated dynamic changes in synchronization index/order parameter, R, measures the level of synchrony for all circadian neuron subgroups using a 1-d rolling window. Comparative differences in the calculated dynamic changes in synchronization index/order parameter, R, in control LD conditions for all neurons in brains cultured with compound eyes (dotted traces), and brains without compound eyes (solid traces). ***D***, Statistical comparisons of overall synchrony between brains with or without compound eyes under control LD conditions. Difference in order parameter, R, between brains with or without eyes were calculated using a randomization analysis (black trace). Dark and gray zones indicate 95% and 99% confidence bands, respectively, under the null hypothesis that there is no difference in order parameter, R, between brains with or without compound eyes attached under control LD conditions; values that fall outside the dark band are statistically significant.

While CRY is the primary light input channel for circadian neurons, opsin-based external photoreceptors contribute a secondary light input channel (Hall, 2005). We measured PER cycling in the circadian circuit under simulated day/night using the LD strobe protocol in whole-brain explants with the eyes attached compared with no eyes to determine whether the presence of the compound eyes, along with CRY, alters circuit cycling. Comparison between the averaged bioluminescence of all circadian cells for brains with or without compound eyes reveals qualitatively no difference in PER oscillations throughout simulated day/night light cycles for a week (Fig. 3*A*, white shade). PER cycles in brains with compound eyes attached appear to dampen more slowly in DD (Fig. 3*A*, blue dotted trace, gray shade vs blue solid trace, gray shade). Additionally, PER oscillations in circadian neurons in brains with compound eyes attached or absent are qualitatively similar (Fig. 3*B*). Quantitatively, the calculated order parameter R between the oscillations of PER in brains with or without the compound eye are similar under simulated day/night cycles but appear slightly more robust in DD conditions (Fig. 3*C*, dotted and solid trace, respectively, DD represented by gray shade). We find no significant differences in calculated R values in PER cycling in cultured brains with and without compound eyes attached during simulated day/night cycles, but under DD, with eyes is significantly higher relative to without eyes (Fig. 3*D*). The similarities in oscillator rhythmicity and phase coherence found in CTRL LD in brains without eyes (Movie 2, left) are compared with CTRL LD in brains with eyes (Movie 3).

Movie 3. Raw time-lapse recordings of adult XLG-PER-Luc *Drosophila* whole-brain explants comparing bioluminescence of brains with and without compound eyes in CTRL LD. Left, Three whole-brain culture explants with compound eyes attached maintained in control conditions (LD strobe with no phase shift) for 9 d followed by 2 d of DD. Right, Three whole-brain culture explants with compound eyes removed maintained in control conditions (LD strobe with no phase shift) for 9 d followed by 2 d of DD. See Materials and Methods for more details. 10.1523/JNEUROSCI.3074-19.2021.video.3

### LL immediately causes loss of per oscillations throughout the circadian circuit

**Figure 4.**
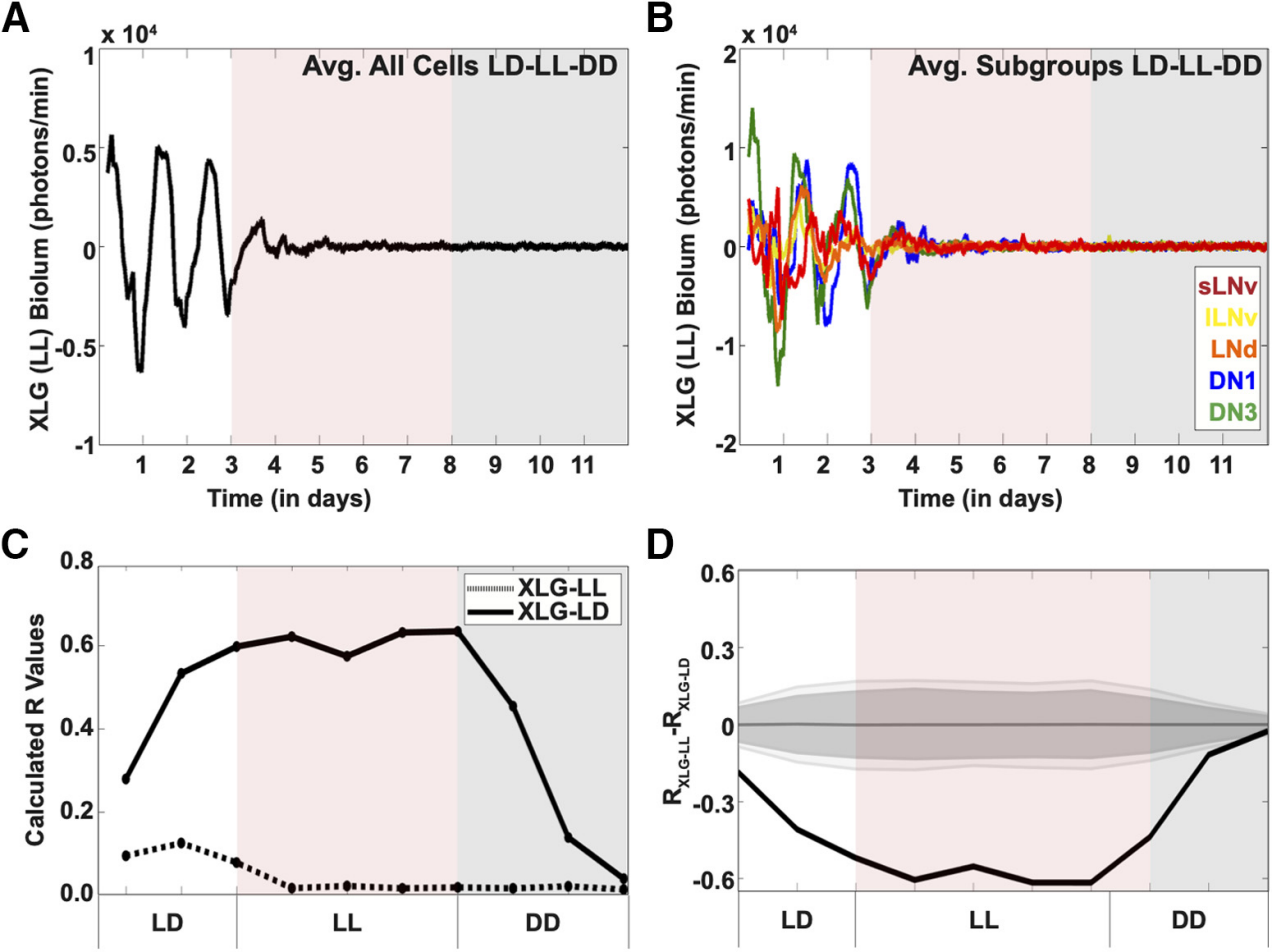
Exposure to LL dampens PER oscillations in *Drosophila* clock neurons. Bioluminescence recordings of XLG-PER-Luc in cultured adult *Drosophila* brains (*n* = 6 brains) under 3 d of control LD, followed by 5 d of LL (red shade), then DD (gray shade). ***A***, Averaged bioluminescence traces of all PER-expressing circadian neurons under 3 d of control LD, followed by 5 d of LL conditions (black trace, red shade) followed by DD (black trace, gray shade). ***B***, Averaged bioluminescence traces of PER expression in circadian neuron subgroups under control LD-LL-DD conditions. Each neuron subgroup is labeled as follows: s-LNv (red), l-LNv (yellow), LNd (orange), DN1 (blue), and DN3 (green). ***C***, Calculated synchronization index/order parameter, R values for PER oscillations (dotted trace) under LD-LL-DD conditions. XLG-PER-Luc (solid trace), under control conditions is overlaid for comparison (s-LNv = 18 cells, l-LNv = 19 cells, LNd = 18 cells, DN1 = 27 cells, DN3 = 18 cells). ***D***, Statistical comparisons of overall synchrony of PER expression under control LD followed by DD and LD-LL-DD conditions. Difference in order parameter, R, between oscillations of PER in control LD or LD-LL-DD were calculated using a randomization analysis (black trace). Dark gray and light gray zones indicate 95% and 99% confidence bands, respectively, under the null hypothesis that there is no difference in order parameter, R, between the oscillations of PER under control LD conditions followed by DD and LD-LL-DD; values that fall outside the dark band are statistically significant.

LL rapidly evokes circadian behavioral arrhythmicity *in vivo* (Pittendrigh and Daan, 1976). However, the detailed linkage between LL-induced changes in PER cycling in the circadian circuit and behavioral arrhythmicity is unknown. Before LL, we imaged cultured whole brains under standard LD strobe to simulate day/night cycles for 3 d before transitioning to 5 d of LL, then into DD (Fig. 4*A*, white, red, and gray shade, respectively). Upon LL, averaged PER oscillations from the entire circadian circuit rapidly dampens and lack of rhythmicity persists well into DD (Fig. 4*A*, red and gray shade, respectively). LL-induced averaged PER oscillations and amplitudes are dampened for all circadian subgroups (Fig. 4*B*); however, the lateral neurons immediately lose oscillations under LL, while DN1 and DN3s have oscillations that weakly persist days after the start of LL (Fig. 4*B*, light red shade). The long-lasting detrimental effects of LL on PER cycling throughout the circadian circuit continues from the transition from LL to DD (Fig. 4*A*,*B*, gray shade). Quantitative analysis of the LD-LL-DD transitions show that synchrony of cells within the circuit under an LL environment rapidly decreases as quantified by the order parameter R (Fig. 4*C*, dotted trace) compared with the overall circuit of brains in a control LD environment (Fig. 4*C*, solid trace), with statistically significant differences between the two conditions (Fig. 4*D*). These results further validate the correspondence between circadian circuit wide PER cycling imaged in whole brain to *in vivo* circadian behavior as both dampen almost immediately in response to LL.

### LL induced per arrhythmicity is partially rescued mutant flies that lack functional CRY in *Drosophila* whole brains

**Figure 5.**
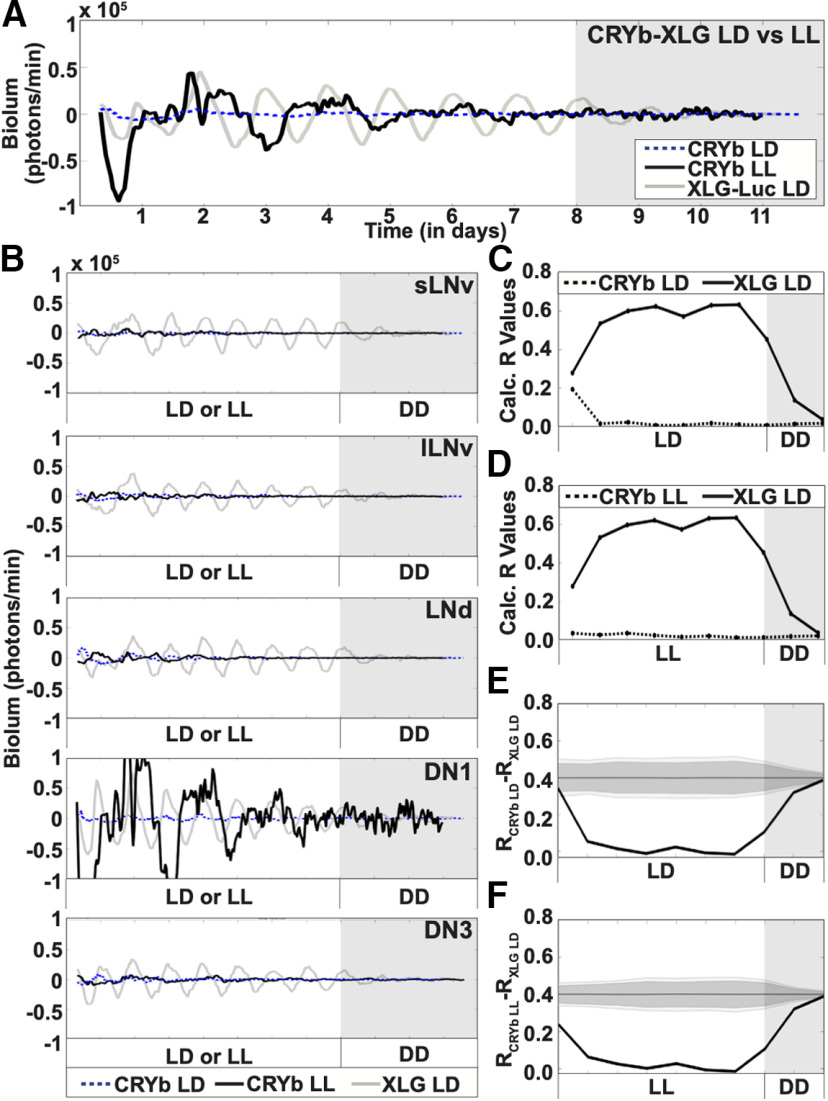
CRY is required for LD entrainment of cultured fly brains. Bioluminescence recordings of *cry^b^* XLG-PER-Luc in cultured adult *Drosophila* brains under 8 d of control LD (*n* = 3 brains; blue dotted trace) and 8 d of LL (*n* = 3 brains, black solid trace), followed by DD (gray shade). XLG-PER-Luc bioluminescence (gray trace) is overlaid for comparison of oscillations between the two genotypes (s-LNv = 18 cells, l-LNv = 19 cells, LNd = 18 cells, DN1 = 27 cells, DN3 = 18 cells). ***A***, Averaged bioluminescence traces of all PER-expressing circadian neurons in a *cry^b^* background under 8 d of control LD (blue dotted trace; s-LNv = 17 cells, l-LNv = 20 cells, LNd = 11 cells, DN1 = 13 cells, DN3 = 25 cells) or LL (black solid trace), followed by DD (gray shade). XLG-PER-Luc expression is overlaid for comparison of the two clock proteins. ***B***, Averaged bioluminescence traces of PER expression in circadian neuron subgroups in a *cry^b^* background under control LD (blue dotted trace) and LL conditions (black solid trace). Each neuron subgroup undergoes both LD and LL conditions (s-LNv = 29 cells, l-LNv = 20 cells, LNd = 16 cells, DN1 = 30 cells, DN3 = 29 cells). XLG-PER-Luc bioluminescence (gray trace) is overlaid for comparison of oscillations between the two genotypes. ***C***, Calculated synchronization index/order parameter, R values for PER oscillations in *cry^b^*-XLG-Luc under control LD conditions (dotted trace). XLG-PER-Luc (solid trace), under control conditions is overlaid for comparison. ***D***, Calculated synchronization index/order parameter, R values for PER oscillations in *cry^b^*-XLG-Luc under LL conditions (dotted trace). XLG-PER-Luc (solid trace), under control conditions is overlaid for comparison. ***E***, Statistical comparisons of overall synchrony between PER from *cry^b^*-XLG-Luc and PER from XLG-Luc under control LD conditions followed by DD. Difference in order parameter, R, between oscillations of PER from *cry^b^*-XLG-Luc under control LD and PER from XLG-Luc from control LD conditions were calculated using a randomization analysis (black trace). Dark gray and light gray zones indicate either 95% or 99% confidence zones, respectively. Here, the null hypothesis indicates that there is no difference in order parameter, R, between the oscillations of PER from *cry^b^*-XLG-Luc and PER from XLG-Luc under control LD conditions. ***F***, Statistical comparisons of overall synchrony between PER from *cry^b^*-XLG-Luc and PER from XLG-Luc under LL and control LD conditions, respectively, followed by DD. Difference in order parameter, R, between oscillations of PER from *cry^b^*-XLG-Luc under LL and PER from XLG-Luc from control LD conditions were calculated using a randomization analysis (black trace). Dark gray and light gray zones indicate 95% and 99% confidence bands, respectively, under the null hypothesis that there is no difference in order parameter, R, between the oscillations of PER from *cry^b^*-XLG-Luc under LL and PER from XLG-Luc under control LD conditions; values that fall outside the dark band are statistically significant.

**Figure 6.**
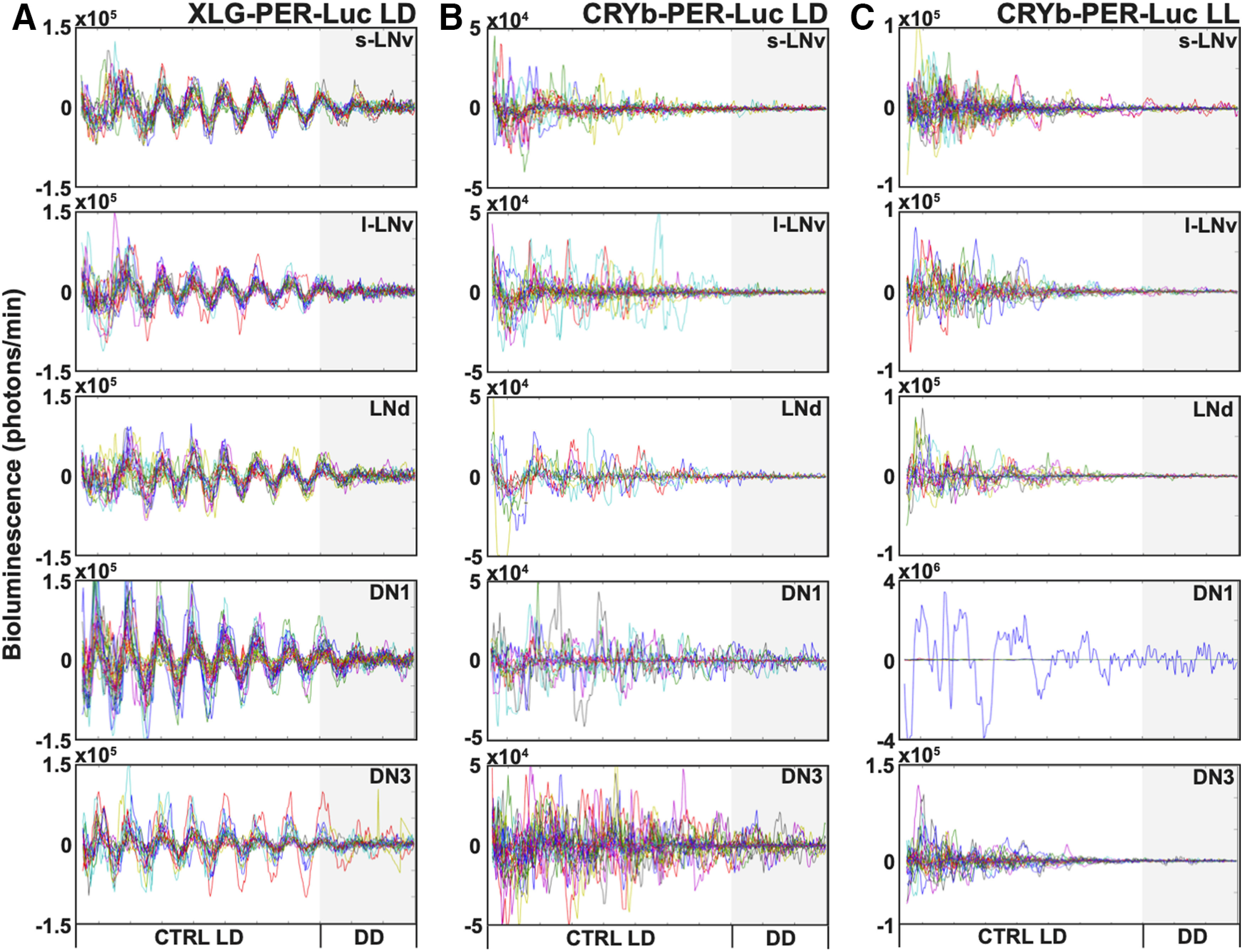
The absence of CRY disrupts synchronized rhythms of individual oscillators under LD and LL measured in different cell groups of cultured fly brains. Bioluminescence recordings showing individual oscillations in cultured adult *Drosophila* brains under 8 d of control LD (*n* = 3 brains) or 8 d of LL (*n* = 3 brains), both followed by DD. ***A***, XLG-PER-Luc individual oscillations (one colored trace indicates one cell) are highly synchronized in 8 d of LD, then gradually dampen in DD (s-LNv = 18 cells, l-LNv = 19 cells, LNd = 18 cells, DN1 = 27 cells, DN3 = 18 cells). ***B***, *cry^b^* XLG-PER-Luc individual oscillations (one colored trace indicates one cell) are high amplitude but desynchronized in 8 d of LD and in following DD (s-LNv = 17 cells, l-LNv = 20 cells, LNd = 11 cells, DN1 = 13 cells, DN3 = 25 cells). ***C***, *cry^b^* XLG-PER-Luc individual oscillations (one colored trace indicates one cell) are detectable but desynchronized in 8 d of LD and in following DD, with a single DN1 cell showing measurable oscillation (s-LNv = 29 cells, l-LNv = 20 cells, LNd = 16 cells, DN1 = 30 cells, DN3 = 29 cells).

CRY plays a crucial role in environmental light entrainment *in vivo* (Emery et al., 1998; Stanewsky et al., 1998). LL exposure disrupts the circadian clock in many animal species, including *Drosophila*, as shown by light intensity-dependent behavioral arrhythmicity (Pittendrigh and Daan, 1976; Emery et al., 2000). In contrast, mutant *cry^b^* and *cry^01^* flies are behaviorally rhythmic in LL, indicating CRY is the primary light input channel for circadian neurons (Dolezelova et al., 2007; Emery et al., 2000). We measured the whole-brain light response of PER cycling throughout the circadian circuit in the functional absence of CRY using XLG-PER-Luc in a *cry^b^* mutant background transgenic line (Harper et al., 2017) under LD and LL conditions. PER oscillations of *cry^b^* mutants in LD and LL with control XLG-PER-Luc in CTRL LD (Fig. 5*A*, blue dotted, black solid, and gray solid lines, respectively). Under LD conditions, *cryb* mutant PER oscillations are completely dampened (Fig. 5*A*, blue dotted trace). In contrast, cry^b^ PER oscillations under LL entrainment exhibit weak oscillations and lower peak bioluminescence (Fig. 5*A*, black solid trace) as compared with those seen in control XLG-PER-Luc under standard LD entrainment (Fig. 5*A*, gray solid trace). In DD following either LD or LL, both *cry^b^* conditions show little or no oscillations (Fig. 5*A*, gray shade). The circadian neurons of *cry^b^* mutants show dampened PER oscillations throughout the entirety of both LL or LD entrainment (Fig. 5*B*) except for the apparent high amplitude oscillations measured in the DN1 subgroup (Fig. 5*B*, black solid trace, fourth panel) under LL conditions. We analyzed this further and find that the DN1s actually tend to be less rhythmic than other groups in cryb flies. A single very large fluctuation in the DN1 sample under LL gives the summed trace a misleading appearance (Fig. 6*C*). In contrast to the high percentage of rhythmic cells in XLG-PER-Luc flies in LD (Fig. 6*A*), the percent rhythmic cells in cryb flies under LD are 12% for s-LNv (*n* = 17 cells), 30% for l-LNv (*n* = 20), 36% for LNd (*n* = 11), 17% for DN1 (*n* = 12), and 31% for DN3 (*n* = 26), using the Lomb-Scargle periodogram applied to 4-d segments (Fig. 6*B*). The percent rhythmic cells in cryb flies under LL are 34% for s-LNv (*n* = 29), 35% for l-LNv (*n* = 20), 31% for LNd (*n* = 16), 20% for DN1 (*n* = 30), and 38% for DN3 (*n* = 29; Fig. 6*C*). We calculated the order parameter R of *cry^b^* PER oscillations under LD (Fig. 5*C*, dotted trace) and LL conditions (Fig. 5*D*, dotted trace) and compared these values to R calculated for control XLG-PER-Luc under LD (Fig. 5*C*,*D*, solid traces) as entrainment progresses into DD (Fig. 5*C*,*D*, gray shade). As expected, PER oscillations in *cry^b^* mutants show no significant synchrony under either LD (Fig. 5*E*) conditions compared with XLG-PER-Luc oscillations. PER oscillations in cry^b^ mutants under LL differs significantly from control XLG-PER-Luc under LD (Fig. 5*F*).

### Exposure to WLSs dampen circadian circuit-wide rhythmicity and synchrony

**Figure 7.**
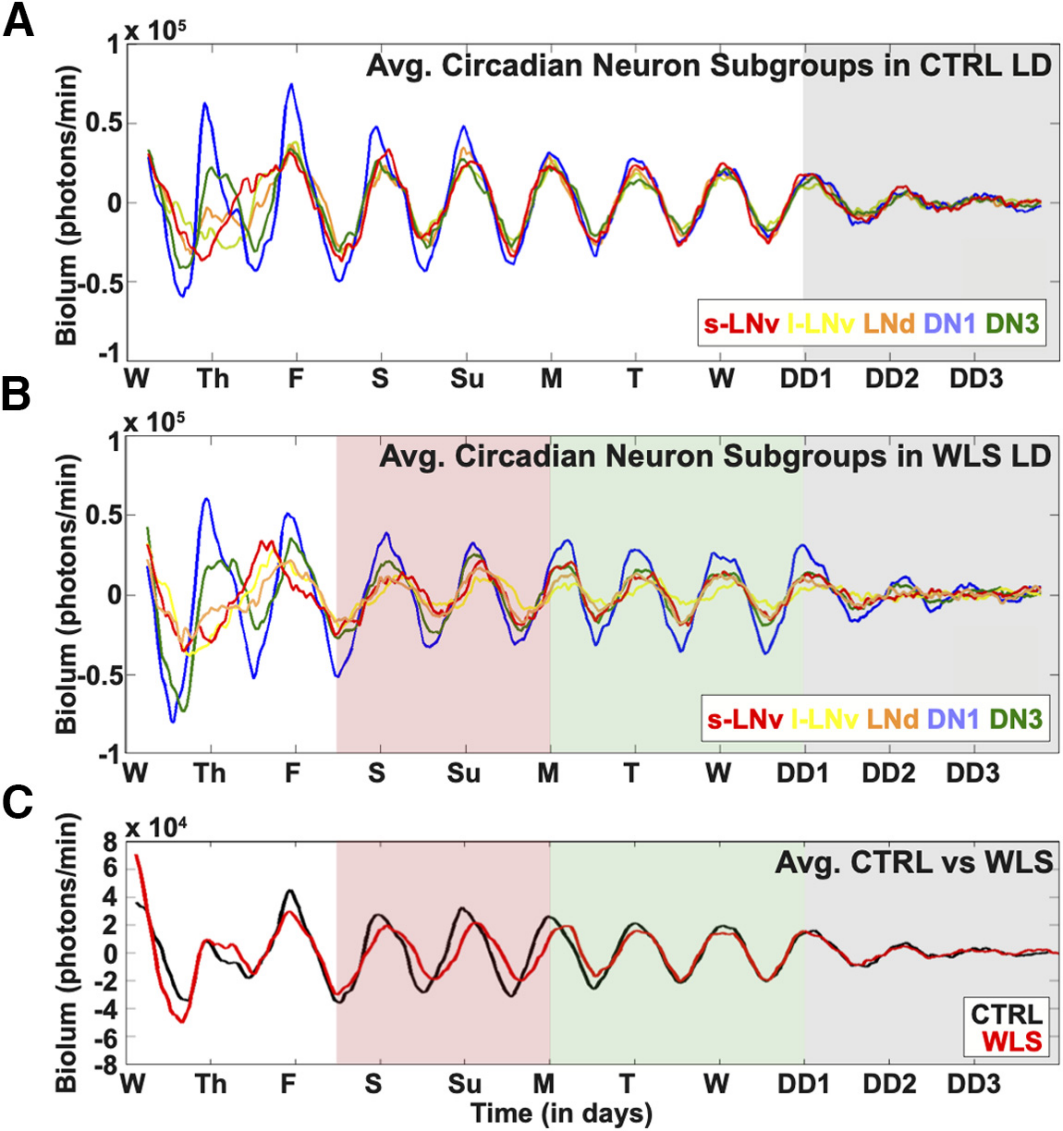
WLSs dampen circuit-wide rhythmicity and synchrony of *Drosophila* circadian neurons. Eleven-day bioluminescence recordings of cultured *Drosophila* brains reported by PER-Luciferase (*n* = 6 brains). ***A***, Control LD strobe conditions simulate standard 12/12LD spanning 8 d, without phase-shifting light signals [s-LNv (red) = 18 cells, l-LNv (yellow) = 19 cells, LNd (orange) = 18 cells, DN1 (blue) = 27 cells, DN3 (green) = 18 cells]. After the one-week simulation, brains are placed in DD to challenge the free-running clock (gray shade). ***B***, WLS conditions subject cultured adult *Drosophila* brains to one simulated weekend entailing a 3-h phase delay on Fridays, which persist until Sunday (red shade). Weekends are followed by a 3-h phase advance to simulate the return to a weekday schedule (green shade) and DD [gray shade; s-LNv (red) = 17 cells, l-LNv (yellow) = 17 cells, LNd (orange) = 15 cells, DN1 (blue) = 28 cells, DN3 (green) = 30 cells]. ***C***, Averaged bioluminescence traces comparing all cells placed in control LD (black trace) or WLS conditions (red trace). Light shifts are indicated as follows: weekend phase delay (red shade), weekday phase advance (green shade), and DD (gray shade). Tick marks on the horizontal axis mark time of lights on for the indicated day under the control LD cycle.

We examined the circadian circuit-wide dynamic response at the single-neuron resolution to compare unshifted to shifted light schedules during simulated daytimes. For 11 d, we obtained bioluminescence imaging of cultured adult *Drosophila* brains exposed to the LD strobe entrainment schedule. Under control (CTRL), unshifted LD conditions, we find all major circadian neuron subgroups exhibiting a synchronized peak of PER-luc signal just before lights on at Friday morning that remains highly synchronous for ∼24 h high amplitude cycles under LD strobe for 7 d until slowly dampening during 3 d of DD (Fig. 7*A*). For the initial period from Wednesday morning through late Thursday evening, PER-luc oscillations are less coherent, particularly for the s-LNv, l-LNv, and the LNds, while the DN1 and DN3 appear more coherent during the initial period. This suggests the whole-brain preparation initially disrupts oscillator amplitude and synchrony differentially between circadian cell groups. After 8 d of unshifted LD, we challenged the free-running clock by placing brains in DD. Clock cycling amplitude dampens in DD (Fig. 7*A*, gray shade).

For WLS within the LD strobe light protocol, we performed light phase shifts by initiating a 3-h phase delay, simulating “staying up late on Friday.” We retained this 3-h phase delayed schedule for 2 d simulating “sleep in late, stay up late” followed by a return to the original entrainment schedule by a 3-h phase advance to simulate “Monday morning.” We find that *Drosophila* whole-brain explants exposed to WLS schedules show reduced synchrony between and within circadian neuron subgroups during (Fig. 7*B*, red shade) and after simulated weekend phase shifts (Fig. 7*B*, green shade). Furthermore, the s-LNv and l-LNv circadian neuron subgroups following WLS show an immediate loss in rhythmicity and synchrony during the transition into DD (Fig. 7*B*, green shade), revealing effects that persist temporally beyond acute light shifts on circadian neural circuit perturbation 3 d after the last phase shift that simulates one weekend. This contrasts with the higher level of synchrony seen in DD for unshifted CTRL (Fig. 7*A*, gray shade). Averaged circuit-wide cycling (Fig. 7*C*) is compared between unshifted CTRL (black) and 3 h WLS (red), indicating average oscillator phase recovery does not occur until several days postshift. Similar weekday/weekend schedules, as previously described, are experienced in a chronic manner by many individuals. For the WLS experimental group, similar to controls, PER-luc oscillations are also less coherent for the initial period from Wednesday morning through late Thursday evening and s-LNv do not appear to be as tightly synchronized with other cell groups just before lights on before Friday, again suggesting the whole-brain preparation is a source of some variance during the first 2 d of the experiment because of differentially disrupted oscillator amplitude and synchrony between groups.

The striking differences in oscillator rhythmicity and phase coherence found in CTRL LD (Movie 2, left) are observed when compared with brains exposed to WLS (Movie 2, right). Phase ensemble animations aid to visualize oscillator dynamics comparing brains in CTRL (left) and WLS (right) schedules averaged as a whole throughout the duration of the experiment (Movie 4), separated into circadian neuron subgroups (Movie 5), and at single-cell level (Movie 6). Differences in intersubgroup dynamics in CTRL and WLS conditions led us to investigate circadian cycling of individual circadian neuron subgroups at single-cell resolution.

Movie 4. The animations show changes in the relative phase and amplitude of XLG-PER-Luc bioluminescence activity for all cells (averaged from all neuronal subgroups) in either control (left) or WLS (right) conditions. Mean network phase is standardized so that the mean network phase is set to ZT 0 on day 3 when entrainment is most stable. The angle of the disks represents the relative phase shift over time such counterclockwise movement indicates a phase delay whereas clockwise movement indicates a phase advance. The drift of the disks toward the center of the circle and the size of the disks indicates reduction in amplitude. See Materials and Methods for more details. 10.1523/JNEUROSCI.3074-19.2021.video.4

Movie 5. The animations show dynamic changes in relative phase shifts and amplitude for each neuronal subgroup in either control conditions (left) or in response to WLS (right). The mean phase shift for each neuron subgroup is represented by polar angle of the disks whereas amplitude is represented by the size and proximal distance of the disks from the center of the circles. The disks are colored according to neuronal subgroup for the s-LNvs (red), l-LNvs (yellow), LNds (orange), DN1s (blue), and DN3s (green). 10.1523/JNEUROSCI.3074-19.2021.video.5

Movie 6. The animations show changes in the phase and amplitude of XLG-PER-Luc bioluminescence activity for individual neuron oscillators from all neuronal subgroups in either control conditions (left) or in response to WLS (right). The angle of the disks represents oscillator phase and drift of the disks toward the center of the circle and the size of the disks indicates reduction in amplitude. The disks are colored according to neuronal subgroup for the s-LNvs (red), l-LNvs (yellow), LNds (orange), DN1s (blue), and DN3s (green). 10.1523/JNEUROSCI.3074-19.2021.video.6

### Individual circadian neuron subgroups exhibit distinct dynamics of activity under WLS conditions

**Figure 8.**
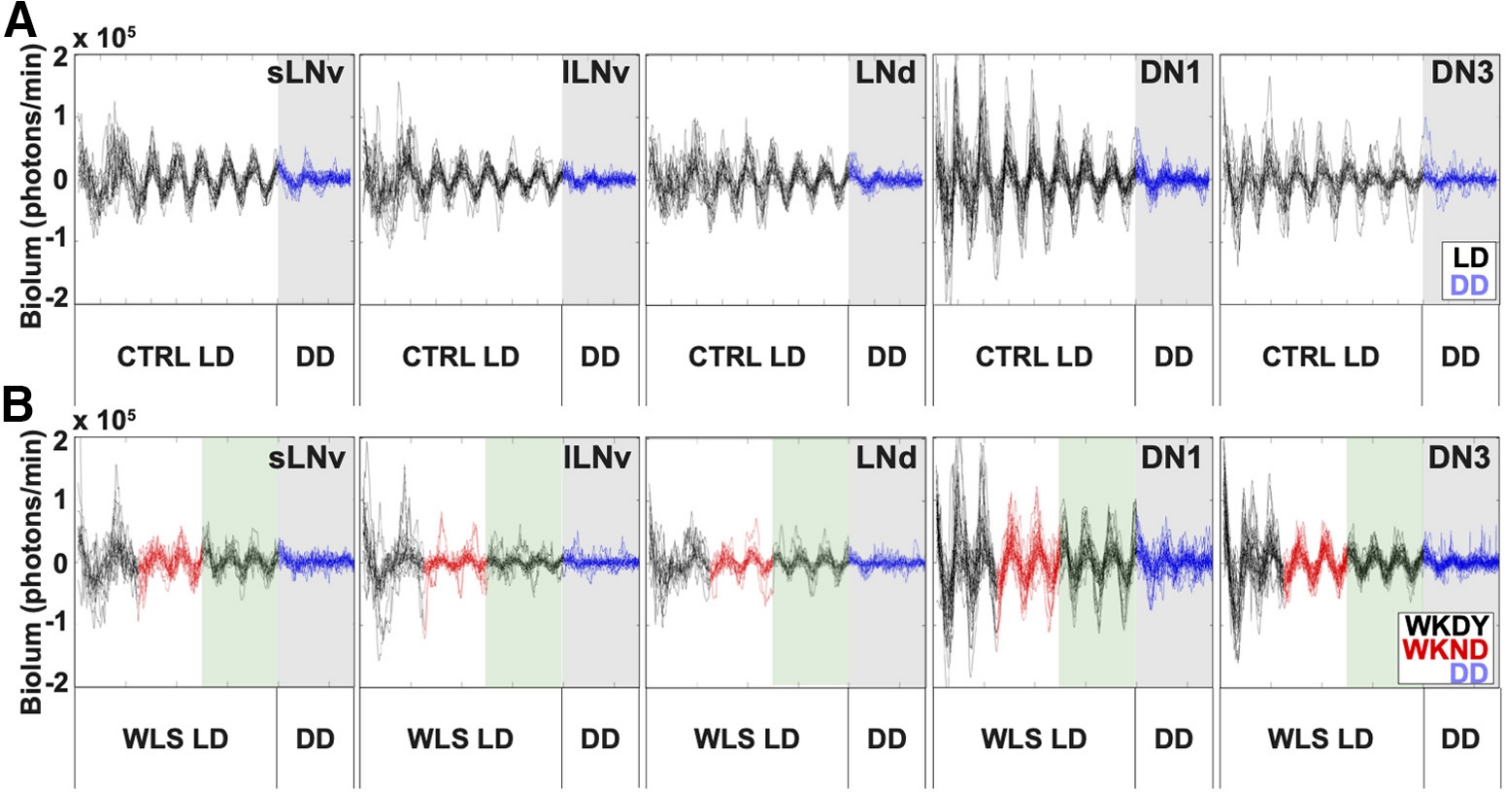
Circadian neuron subgroups exhibit distinct dynamics of activity in response to WLS conditions. Bioluminescence traces for individual cells from each circadian neuron subgroup. Each trace in one panel represents bioluminescence from one individual cell. ***A***, Bioluminescence traces from brains subjected to control LD without phase advancing light signals for 8 d (black traces), then placed in DD conditions (blue traces, gray shade; s-LNv = 18 cells, l-LNv = 19 cells, LNd = 18 cells, DN1 = 27 cells, DN3 = 18 cells). ***B***, Bioluminescence traces from brains subjected to WLS LD conditions. WLS conditions entail pre-WLS (black trace, white shade), WLS (red trace, white shade), post-WLS (black trace, green shade), followed by DD (blue trace, gray shade; s-LNv = 17 cells, l-LNv = 17 cells, LNd = 15 cells, DN1 = 28 cells, DN3 = 30 cells).

**Figure 9.**
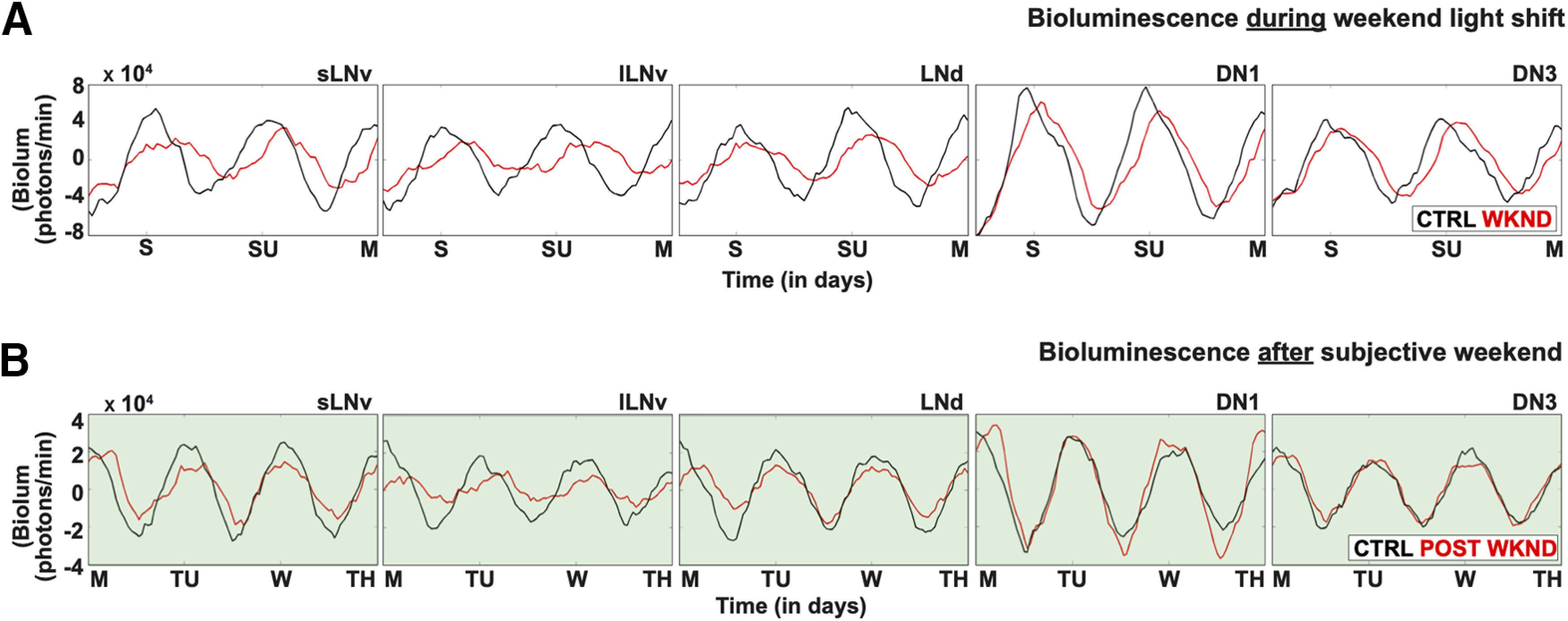
Detailed traces of averaged bioluminescence during and postexposure to WLS. ***A***, Averaged bioluminescence traces for each circadian subgroup comparing the “simulated weekends” in control LD (black trace) with WLS conditions (red trace). ***B***, Averaged bioluminescence traces for each circadian subgroup comparing the “post-WLS” weekdays in control LD (black trace) and WLS conditions (red trace). Traces for both control LD and WLS conditions were generated using custom MATLAB scripts. See Materials and Methods for more details.

Under unshifted CTRL LD, circadian neuron subgroups exhibit distinct signatures in rhythmicity, phase coherence and amplitude throughout the duration of entrainment (Fig. 8*A*, black traces), which dampen in DD (Fig. 8*A*, blue traces against gray shaded background). The s-LNvs show the highest degree of synchrony over the course of CTRL LD (Fig. 8*A*, first column). The DN1s exhibit the highest amplitude oscillations (Fig. 8*A*, fourth column), and the DN3s exhibit the greatest variability in oscillator amplitude and synchrony in CTRL LD (Fig. 8*A*, fifth column). WLS disrupts rhythmicity and synchronization during (red traces) and after (black traces on green shaded background) shifts affecting all circadian neuron subgroups except for DN3s (Fig. 8*B*), which significantly tighten their amplitude and phase coherence (Fig. 8*B*). Interestingly, DN3s are the only cells that become more synchronized in response to WLS returning to a weekday schedule (Fig. 8*B*, fifth column, green shaded background). Averaged waveforms for the s-LNv, l-LNv, LNd, DN1 and DN3 are shown during the WLS on Saturday, Sunday, and Monday (Fig. 9*A*) and post-WLS on Monday, Tuesday, Wednesday, and Thursday (Fig. 9*B*).

**Figure 10.**
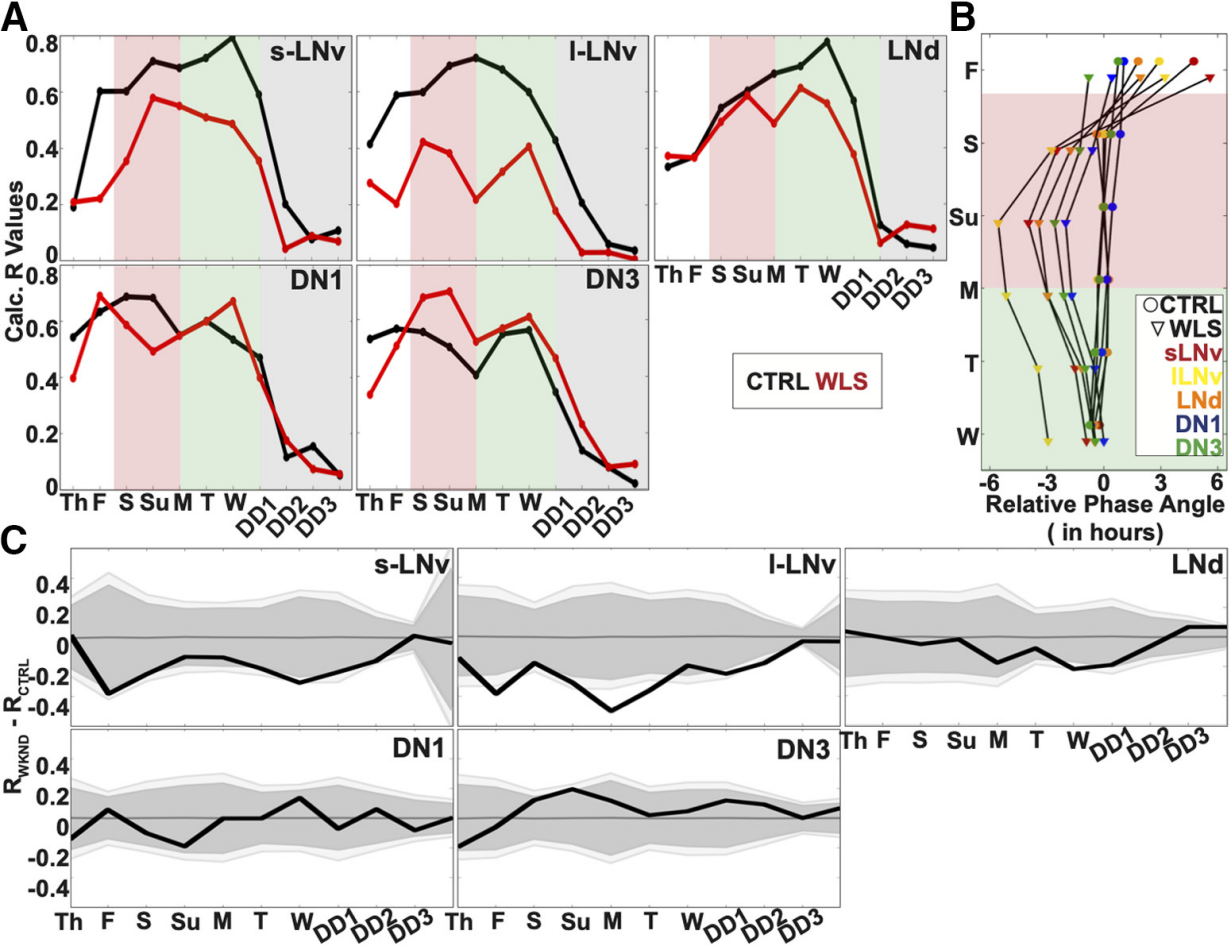
Circadian neuron subgroup synchrony exhibit destabilization in dynamics during WLS and requires days to recover postshift. Calculated dynamic changes in synchronization index/order parameter, R, measures the level of synchrony for each circadian neuron subgroups using a 1-d rolling window. ***A***, Calculated order parameter, R, comparing LD conditions between Control LD with no shifts (black) and WLS LD with 3-h shifts (red). WLS schedule is marked during the following light schedules: preshift (white shade), WLS (red shade), postshift (green shade), and DD (gray shade). ***B***, Comparison of phase angle changes between each circadian neuron subgroups under control LD (circle) and WLS conditions (triangle). WLS conditions are indicated as follows: preshift (white shade), WLS (red shade), postshift (green shade). Each circadian neuron subgroups for control LD and WLS conditions are as follows: s-LNv (red), l-LNv (yellow), LNd (orange), DN1 (blue), DN3 (green). ***C***, Statistical comparisons of subgroup synchrony between control LD and WLS conditions followed by DD. Difference in order parameter, R, between control and WLS (RWLS-RCTRL) conditions were calculated using a randomization analysis (black trace). Dark and gray zones indicate 95% and 99% confidence bands, respectively, under the null hypothesis that there is no difference in order parameter, R, between brains placed in control LD or WLS LD; values that fall outside the dark band are statistically significant.

Quantitatively, s-LNvs and LNds maintain the highest level of synchrony in CTRL conditions as shown by the increasing order parameter R values in simulated LD day/night (Fig. 10*A*, black lines). In DD, following CTRL LD under LD strobe, all neuronal subgroups exhibit a clear and immediate decrease in phase, period and coherence as quantified by order parameter R (gray shaded background). Small-LNvs and large-LNvs show large changes in relative phase angle (Fig. 10*B*) and significantly lower synchrony in response to WLS relative to unshifted CTRL LD (Fig. 10*A*,*C*). Conversely in response to WLS, LNds, DN1s, and DN3s maintain relatively more robust synchrony and amplitude as measured by order parameter R (Fig. 10*A*, red traces), smaller changes in relative phase angle (Fig. 10*B*), and few significant differences between control and shifted light conditions (Fig. 10*C*). Despite being cell autonomously light-blind by not expressing CRY or Rh7 like other subgroups (Benito et al., 2008; Yoshii et al., 2008; Ni et al., 2017), DN3s show significantly increased intergroup oscillator synchrony during the weekend as a response to WLS relative to unshifted CTRL LD (Fig. 10*A*,*C*, see also Fig. 8*A*,*B*, far right panels). Under both CTRL LD and WLS, phase synchrony gradually improves for the first few days in culture, which may be a potential confounding factor for different groups of neurons. The DN3s of WLS brains before the WLSs are closer to the phase they have to reach during WLS relative to other circadian groups (Fig. 10*B*) and this may be because of the relatively short “settling into synchrony” first few days of the measurements. However, we see in Figure 10*C* that there are no statistically significant differences for any of the cell groups for day before the shift except for the DN3s which are closest to the phase they have to reach during WLS. WLS evokes increased DN3 synchrony between Friday through Tuesday, going from worse than the control to better than the control. It is possible that such a re-synchronization is enabled by the lack of synchrony in other cell groups. However, this is not entirely clear as the relative lack of synchrony in other cell groups during the first few days in culture does not evoke a similar improvement in DN3 synchrony before the light shifts. It is also worth noting that, while the order parameter R is calculated using 1-d segments, the phase angles shown in Figure 10*B* are calculated using 2-d segments, resulting in the apparent discontinuity between the tight alignment of averaged peaks for all cell groups on Friday as shown in Figure 7*A* for CTRL LD and the apparently larger relative phase angles for the CTRL LD for the s-LNv reported in Figure 10*B*. To express the dynamic complexity of the circadian circuit-wide response to WLS, animations are provided (Movies 4, 5, 6).

### Exposure to WLS leads to sleep disruption and defects in learning and memory

**Figure 11.**
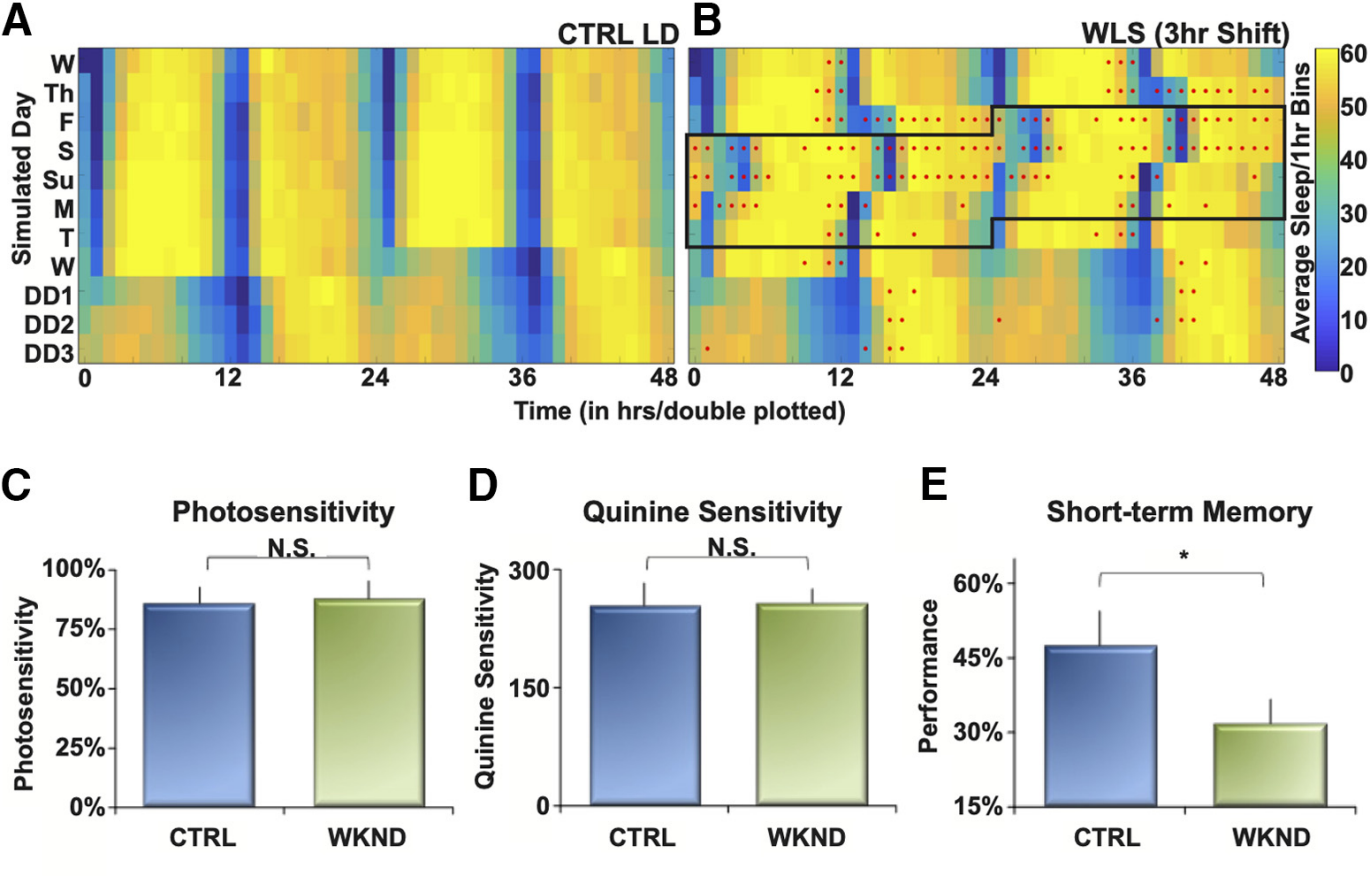
WLS leads to sleep disruption and hinders learning and memory. ***A***, Double-plotted heat map indicates the average amount of sleep of whole, intact flies per 1-h bins under control LD conditions. Maximum amount of sleep/1-h bin is shown in yellow, and no sleep (0 min) is shown in blue. Each row represents elapsed time: 2 d, 48 h for double plotting. Control LD includes 8 d of simulated daytime and nighttime followed by 3 d of DD (*n* = 64 flies). ***B***, Double-plotted heat map indicating sleep amount of whole, intact flies under a WLS LD schedule. Maximum amount of sleep/1-h bin is shown in yellow, and no sleep (0 min) is shown in blue. WLS schedule entails a 3-h phase delay during simulated weekends followed by a 3-h phase advance simulating return to a weekday schedule. Flies are subjected to DD after 8 d of LD. Red dots indicate significant differences in sleep/1-h bin between control LD and WLS LD (*n* = 64 flies). ***C–E***, APS assay was used to determine how WLS affects learning and memory. (***C***) Photosensitivity assay comparing flies exposed to control LD (blue) and WLS conditions, 2 d after the 3-h phase advance (green; *n* = 5 male flies/condition). ***D***, Quinine sensitivity measurements for flies placed in either control LD (blue) and WLS conditions (green) 2 d after the 3-h phase advance (*n* = 5 male flies/condition). ***E***, Measurement of learning and memory for flies exposed to WSL using the APS assay. Performance is measured for fly ability to remember to avoid specific parts of a T-maze 2 d after exposure to an LD condition. Control LD flies are indicated in blue, post-WLS flies in green (*n* = 10–11 male flies/condition). Significance for differences between control LD and WLS LD was determined using a *t*-test, *p* ≤ 0.05 indicated by *, *p* > 0.05 is not significant (N.S.).

To assess potential consequence of the postshift changes in oscillator ensemble activity in circadian neurons following WLS, we measured correlative behavioral outputs under the similar light shift protocols *in vivo*. We exposed whole, intact flies to CTRL LD and WLS schedules used in while-brain imaging while measuring sleep (Hendricks et al., 2000; Shaw et al., 2000). Sleep is stable for unshifted CTRL LD (Fig. 11*A*) as shown by consistent amplitudes (*x-axis*) and robust waveform (*y*-axis) across multiple days (*z*-axis). Flies exposed to WLS have significantly disrupted sleep patterns only during and after phase shifts (Fig. 11*B*, red dots indicate significant hourly difference in sleep compared with CTRL LD). Hourly sleep differences between CTRL LD and WLS groups persist for multiple days (Fig. 11*B*). A closer look at sleep shows groups at a number of initial individual time points before and at the 12-h point that there are significant differences between the CTRL LD and WLS groups. If we examine total sleep across each 24 h, the WLS flies get more total sleep following the light shifts, with no significant difference on the first Wednesday, Thursday, and Friday, or the following Wednesday onwards. For the WLS-effected days, there are significant differences in total sleep between the CTRL LD and WLS on Saturday (*p* = 0.0005), Sunday (*p* = 0.009), Monday (*p* = 0.013), and Tuesday (*p* = 0.0205; Fig. 11*B*, red lines indicate significant daily difference in sleep compared with CTRL LD). The apparent initial differences in sleep for certain time points between the two groups may be because of individual variability among flies in the timing of the steep drop from high levels of sleep to low levels near the 12-h mark.

Decreased cognitive performance, including learning and memory, is linked to circadian dysregulation and sleep loss (Stickgold et al., 2001; Donlea et al., 2011). We tested how WLS affects learning and memory using the APS assay (Seugnet et al., 2009) 2 d after the phase-advancing light shift. WLS flies show significant impairments in remembering where to avoid aversive stimuli (quinine) in the maze compared with flies in CTRL LD (Fig. 11*E*). The behavior exhibited suggests that WLS exposure impairs learning and maintenance of STM days following weekend phase shifts as shown by the inability for conditioning to avoid aversive stimuli, coinciding with persistent postshift circadian circuit and sleep pattern impairment.

## Discussion

Well-established work on SPPs inspired us to test fragmented light/dark periods that permit bioluminescence imaging without disruption of the circadian clock (Pittendrigh and Minis, 1964; Pittendrigh and Daan, 1976). Based on the strict criteria of no measurable differences in circadian free running behavior and the consideration that periodic light exposure throughout the day may help maintain the clock circuit, the LD strobe light schedule faithfully replicates standard 12/12 h light/dark cycles. In addition, the LD strobe light schedule provides optimal time frames to maximize light input and acquisition of bioluminescence images. Our whole-circuit imaging using LD strobe provides longitudinal multiday bioluminescence recordings of the entire circadian neural network at single-cell resolution with reporters for PER and TIM. The relative phase peaks of PER and TIM are separated by ∼3 h, consistent with very highly time resolved ICC snapshots for anti-PER and anti-TIM taken at 1-h intervals that show sequential nuclear accumulation of PER and TIM (Shafer et al., 2002). This relative phasing of PER and TIM is one of many independent lines of evidence that the clock reporter cycling in whole brain reflect the *in vivo* circadian clock circuit. In a previous study, we compared a matrix of whole-brain imaging of PER cycles in five different circadian neuronal subgroups (s-LNv, l-LNv, LNd, DN1, and DN3) with six different time-matched light conditions that were strategically chosen to test predictions of PER peaks or troughs across 3 d with anti-PER ICC analysis of brains collected from behaving flies. Our detailed whole-brain dynamic PER cycling results predicted all less time resolved ICC results for the 5 × 6 matrix, with a statistical confidence value of 10^−27^ (Roberts et al., 2015). Furthermore, LL rapidly induces circadian behavioral rhythmicity (Pittendrigh and Daan, 1976; Baik et al., 2018, 2020). Circadian rhythmicity under an LL entrainment is preserved in mutant flies lacking functional CRY (Emery et al., 2000; Dolezelova et al., 2007; Baik et al., 2019). In close agreement with well characterized LL *in vivo* behavioral responses, PER-cycling rapidly dampens throughout the circadian circuit in response to LL exposure in imaged whole brains, thus providing another line of validation for the whole-brain imaging method. In cryb mutants, PER cycling is disrupted relative to controls both in LD and LL, with a single cell in the DN1 group showing cycling in LL. Previous work shows that the DN1s act as output neurons for the circadian circuit (Cavanaugh et al., 2014; Barber et al., 2016). *In vivo* behavioral responses temporally correspond to whole-brain imaged PER cycling responses to LD, thus providing yet another line of validation that the whole-brain imaging method reflects light responses of the circadian circuit. Lastly, light shifts induce disruption of circadian regulated behaviors including sleep; and learning and memory *in vivo* (more below). While the circadian circuit is clearly capable of responding to other Zeitgebers such as temperature or metabolic cues (Hall, 2005), imaged whole-brain PER cycling is highly responsive to light and closely resembles the timing of *in vivo* light responses for all measures tested. However, under DD conditions, whole-brain PER cycling is much less robust than *in vivo* PER cycling (Roberts et al., 2015, 2016), along with other behavioral responses to DD that include restoration of behavioral rhythmicity transitioning from LL to DD *in vivo* (Power et al., 1995).

Under simulated LD cycles, we obtained single cell resolution circadian circuit responses to unshifted regular light that shows highly synchronized circadian network phase in response to alternating light pulses of LD strobe. This indicates that properly timed Zeitgebers at the start and end of daytime hours, with standard night-time darkness are the most critical light input features for proper entrainment, in agreement with earlier studies employing SPP protocols (Pittendrigh and Daan, 1976). In contrast, oscillators exhibit immediate damping in rhythmicity and synchrony when transitioning from LD to DD indicating that steady photoentrainment is critical for maintenance of robust oscillator synchrony and physiological rhythmicity in the whole-brain preparation in contrast to more robust circadian cycling *in vivo* as detected non-dynamically by anti-PER ICC (Zerr et al., 1990; Roberts et al., 2015, 2016).

Different anatomically defined subgroups of circadian neuronal oscillators exhibit differences in timing in response, varying degrees of change in intersubgroup and intrasubgroup synchrony and amplitude and timing of recovery in response the WLS phase delay followed by a light phase advance days later. Single-cell resolution analysis reveals that rhythmicity and synchrony in s-LNvs, l-LNvs, and LNds immediately dampens by initial Friday phase delay to mark the start of WLS. Taking the LD and DD data together, the s-LNvs are the most stable of all circadian subgroups in the absence of light shifts but are most labile in response to changes in light entrainment evoking the “first out, last back in” pattern of phase destabilization and phase retuning (Roberts et al., 2016). In contrast, DN1s while showing light-induced phase shifts, maintain greater rhythmicity and synchrony in response to the phase delays. Earlier work suggests that the DN1s are the only rhythmic neurons in LL when the CRY input pathway is disrupted (Murad et al., 2007; Stoleru et al., 2007). Surprisingly, the DN3s increase their synchrony in response to phase delays in addition to the striking reduction in oscillator amplitude variance. LNds and the light blind DN3s restore rhythmicity faster than other subgroups after exposure to a simulated weekend. Remarkably, the DN3s increase in phase synchrony and amplitude coherence during and after WLS entrainment. This raises the possibility that DN3s might code for the initial time phase before the light induced shift to a new phase, thus acting as a temporal placeholder. The LNds and DN3s may play a critical role in prompting the remaining circadian neural network into a new state of adaptation of the phase-shifted synchrony, consistent with earlier evidence indicating LNds track phase-advance shifts more rapidly than other subgroups (Roberts et al., 2015, 2016).

Light input from external photoreceptors, such as the compound eye, are also sufficient to reset clock entrainment (Helfrich-Förster et al., 2001). Light entrainment in flies lacking single light input channels (CRY or external opsin expressing photoreceptors or Rh7) yields very discernable but slower behavioral light circadian phase shift responses (Helfrich-Förster et al., 2001; Ni et al., 2017), indicating these input channels are largely functionally redundant (although there are measurable differences between the number of transient days needed for reentrainment at different light intensities and morning vs evening reentrainment depending on which light input channel is missing). Electrophysiological light responses can be recorded in circadian neurons using light stimulus parameters that are optimized for opsin activation in eyes but are insufficient in duration (and perhaps amplitude) for CRY activation (Li et al., 2018; see Baik et al., 2019 for detailed light parameters for CRY activation). As expected, we find that overall circuit entrainment by direct light is comparable whether the compound eye is present or is completely removed. This supports the idea that there is functional redundancy between internal cell autonomous photoreceptors and external opsin-based photoreceptors.

The significant loss in amplitude and synchrony between oscillators following WLS relative to CTRL transitioning to DD suggests that effects persist temporally beyond the immediate light shifts and residual circadian circuit instability may be masked by light inputs into the neural circuit. Together, the data show desynchrony of the circadian circuit persisting after phase delays and advances of the WLS weekend. The circadian circuit is likely destabilized for the greater part of the week for individuals that shift every weekend as a matter of lifestyle. Considering the correlative defects in post-WLS sleep stability, learning, and memory, this poses the critical question of whether these defects are cumulative and can be detrimental over time.

Phase shifts because of “jetlag” disrupt the timing of both arousal/wake and sleep. Sleep in mammals and flies share many similar biological features, but do show some qualitative differences including crepuscular organization of sleep/wake bouts in flies and sleep duration (Hendricks et al., 2000; Shaw et al., 2000). Circadian neurons functionally segregate to control arousal (s-LNvs, l-LNvs) versus sleep (DN1s; Parisky et al., 2008; Shang et al., 2008; Sheeba et al., 2008a,b; Guo et al., 2016). *In vivo* luciferase calcium monitoring at circadian neuronal subgroup spatial and temporal resolution confirms that the s-LNv and l-LNv intracellular calcium signaling exhibit biphasic morning and late day peaks corresponding to arousal while a subset DN1s coincide highest intracellular calcium levels with sleep (Guo et al., 2016, 2017). This innovative imaging approach yields robust records of circadian signal transduction occurring in these neurons in the absence of potential contamination from light excitation necessary for fluorescence imaging.

Circadian clocks regulate numerous aspects daily animal physiology and behavior. Light is the primary environmental zeitgeber for circadian entrainment for many animals, including *Drosophila* (Helfrich-Förster et al., 2001) and humans (Czeisler et al., 1999). While we acknowledge that there are other *in vivo* inputs to the circadian circuit, we find that light shift conditions that closely approximate WLS lead to effects that persist temporally beyond acute light shifts on desynchrony in much of the adult *Drosophila* circadian neural network measured at the single-cell resolution for over a simulated week *ex vivo* that strongly coincide with the disruption of circadian regulated behavioral outputs *in vivo*. Adult flies exposed to WLS exhibit transient defects in memory, learning, and sleep stability between 4 and 6 d of the week. This suggests that for weekly repeated WLS, functional consequences downstream to circadian desynchrony may be present during most days of the week. Clock disruption through repeated light shifts may underlie more severe complications because of cumulative weekly repetitions throughout an individual’s life. Based on the many molecular and circuit-circuit organizational similarities between *Drosophila* and mammals, the circadian neural network responses we measure to WLS conditions may be instructive for understanding light shifts in humans and other animals.
